# VICatMix: variational Bayesian clustering and variable selection for discrete biomedical data

**DOI:** 10.1093/bioadv/vbaf055

**Published:** 2025-03-17

**Authors:** Jackie Rao, Paul D W Kirk

**Affiliations:** MRC Biostatistics Unit, University of Cambridge, Cambridge, CB2 0SR, United Kingdom; MRC Biostatistics Unit, University of Cambridge, Cambridge, CB2 0SR, United Kingdom; CRUK Cambridge Centre Ovarian Programme, University of Cambridge, Cambridge, CB2 0RE, United Kingdom; Cambridge Institute of Therapeutic Immunology and Infectious Disease (CITIID), University of Cambridge, Cambridge, CB2 0AW, United Kingdom

## Abstract

**Summary:**

Effective clustering of biomedical data is crucial in precision medicine, enabling accurate stratification of patients or samples. However, the growth in availability of high-dimensional categorical data, including ’omics data, necessitates computationally efficient clustering algorithms. We present VICatMix, a variational Bayesian finite mixture model designed for the clustering of categorical data. The use of variational inference (VI) in its training allows the model to outperform competitors in terms of computational time and scalability, while maintaining high accuracy. VICatMix furthermore performs variable selection, enhancing its performance on high-dimensional, noisy data. The proposed model incorporates summarization and model averaging to mitigate poor local optima in VI, allowing for improved estimation of the true number of clusters simultaneously with feature saliency. We demonstrate the performance of VICatMix with both simulated and real-world data, including applications to datasets from The Cancer Genome Atlas, showing its use in cancer subtyping and driver gene discovery. We demonstrate VICatMix’s potential utility in integrative cluster analysis with different ’omics datasets, enabling the discovery of novel disease subtypes.

**Availability and implementation:**

VICatMix is freely available as an R package via CRAN, incorporating C++ for faster computation, at https://CRAN.R-project.org/package=VICatMix

## 1 Introduction

The process of identifying groups of similar objects in data, known as *cluster analysis*, is an area of research holding many key applications for biological data. For example, in precision medicine, being able to identify subtypes of disease where patients are grouped based on clinical and/or genomic data can allow clinicians to identify optimal treatments for each group and allow for stratified medicine approaches (Sørlie *et al.* 2001, Verhaak *et al.* 2010, Kuijjer *et al.* 2018, Chen *et al.* 2019). Another application of clustering is in microarray analysis, where groups of samples with similar patterns of gene expression can be identified allowing scientists to improve understanding of functional genomics (Medvedovic *et al.* 2004, Dahl and Newton 2007, Crook *et al.* 2019). Further biomedical applications include, but are not limited to, identifying proteins with similar functions (Zaslavsky *et al.* 2016) and analysing health records and surveys (Molitor *et al.* 2010, Ni *et al.* 2019). Clustering is usually performed in an unsupervised manner, and it is important that clusters are reliable in the sense that clusters can be clearly separated and hold stability in other independent datasets. For example, it is important that a disease subtype can be characterized in a way so it may be differentiated from other subtypes where the optimal treatment pathway could be very different.

Popular classical approaches to clustering include algorithmic methods such as k-means (Hartigan and Wong 1979) and hierarchical clustering (Kaufman and Rousseeuw 1990). However, these methods are largely heuristic and have little statistical characterization of the resulting clusters. An alternative view for the clustering problem is by using model-based clustering, where observations are treated as samples from a mixture model distribution. Finite mixture models are mixtures of probability distributions, where each distribution corresponds to a cluster and is given a different weight pertaining to the proportion of observations assigned to the cluster (McLachlan *et al.* 2019). The expectation-maximization (EM) algorithm can be used to perform maximum likelihood estimation for the model parameters (Fraley and Raftery 2002, McLachlan *et al.* 2019) in a finite mixture model with a fixed number of clusters K. Two R packages implementing the EM algorithm for mixture models are *mclust* (Scrucca *et al.* 2023) and *FlexMix* (Leisch 2004), which can both fit a finite mixture of Gaussian distributions using EM, while the latter additionally allows for mixtures of linear regressions, generalized linear models and other frequentist models.

An obstacle to the use of finite mixture models is that prior knowledge of the finite number of components K in the model is needed in order to fit data to the distribution. However, the true number K is not usually known, and determining this number is an important issue (Celeux *et al.* 2018). Frequentist methods of circumventing this issue include performing model selection with information criterion such as the Bayesian Information Criterion (BIC) (Schwarz 1978, Keribin 2000) or the integrated classification likelihood (ICL) (Biernacki *et al.* 2000). Both of these are employed in *mclust* and *FlexMix*. However, these methods require fitting many finite mixture models with a range of K, and may not be appropriate for the clustering problem; e.g. BIC has been shown to underestimate the true number of clusters (Zhao *et al.* 2015).

Alternatively, Bayesian approaches to model-based clustering can allow for the number of clusters to be a parameter for inference (Nobile and Fearnside 2007, Miller and Harrison 2017). One example is the R package *BayesBinMix* (Papastamoulis and Rattray 2017), an implementation of finite mixture models for binary data, which allows K to be a parameter for inference by having a discrete prior over $1:K_{\text{max}}$ to determine the number of clusters in the model. Priors are also placed upon the other parameters in the model, such as the mixing weights and the cluster-specific parameters. Other Bayesian approaches use reversible jump Markov Chain Monte Carlo (RJMCMC) (Richardson and Green 1997, Tadesse *et al.* 2005), moving between mixture models with different values of K, or use ‘overfitted’ mixture models, where K is set to be more than the number of clusters expected, and a sparse Dirichlet prior on the mixing weights encourages emptying of unnecessary clusters (Rousseau and Mengersen 2011, Frühwirth-Schnatter and Malsiner-Walli 2018).

Bayesian approaches also allow for a range of other mixture models. A popular example is the Dirichlet process mixture model (Maceachern 1994, Escobar and West 1995), a Bayesian nonparametric model using a Dirichlet process prior to allow for an infinite number of clusters, where the number of non-empty clusters can be inferred using Markov Chain Monte Carlo (MCMC) samplers, and the number of clusters is allowed to grow unboundedly as the number of observations increases (Müller and Mitra 2013, Wade and Ghahramani 2018). These models have been implemented for the clustering of biological data frequently in the literature—examples of these include an application to health survey analysis (Molitor *et al.* 2010) and in the R package *PReMiuM* (Liverani *et al.* 2015).

By far the most popular method for estimation of the intractable posterior in both the finite and infinite models is MCMC, allowing for (asymptotically) exact samples from the target density (Robert and Casella 2004, Bishop 2006). The most commonly used MCMC approaches for mixture models include Gibbs sampling (Neal 2000), as well as Metropolis-within-Gibbs when non-conjugate priors are used (Bouguila *et al.* 2008). Such samplers have broad applicability to a range of models used across biomedicine, including topic models (Lu *et al.* 2016, El Hachem *et al.* 2023, Tataru *et al.* 2023). To improve mixing, split and merge MCMC moves have also been proposed when performing inference for mixture models (Jain and Neal 2004).

However, there are some major drawbacks concerning the use of MCMC. Retrieving one optimal clustering structure from the MCMC samples which is most representative of the posterior is difficult due to the multimodality of the posterior surface, and there are often mixing issues where chains are prone to being stuck, making it difficult for some MCMC samplers to reach convergence (Celeux *et al.* 2000, van Havre *et al.* 2015, Wade and Ghahramani 2018). There is also the ‘label switching’ phenomenon, where the same clustering structure appears to be different across the MCMC samples as the labels associated with the clusters change (Redner and Walker 1984), requiring algorithms or summarization methods to circumvent this problem. Finally, MCMC algorithms are also generally very computationally expensive; they require a large number of iterations to sufficiently explore the high-dimensional posterior surface, and often are infeasible when datasets are large.

An alternative to MCMC methods is variational inference (VI), which is a deterministic approximation method allowing us to instead turn the inference problem to an optimization problem, by finding an approximation to the posterior. It is consistent for the estimation of mixture models under certain assumptions (Chérief-Abdellatif and Alquier 2018), and it is much more efficient in terms of computational time and memory usage, therefore, allowing us to scale mixture models to larger datasets (Blei *et al.* 2017). Implementations of VI in mixture models perform well in practice on a variety of datasets including in computational biology (Blei and Jordan 2006, Constantinopoulos *et al.* 2006, Guan *et al.* 2010). However, VI is an approximate inference method; solutions are sensitive to initialization and often are stuck in local optima (Bishop 2006). To circumvent this, we utilize a novel method of implementing a co-clustering matrix—similar to posterior similarity matrices used often in post-processing of MCMC output—to average over multiple initializations, while still maintaining efficiency.

Biological data, especially ’omics data, is often high-dimensional where only a subset of the variables are relevant to the inherent clustering structure, motivating the use of methods which allow for variable selection. Methods for the finite mixture model case often include feature selection via the inclusion of latent covariate selection variables to represent feature saliency (Law *et al.* 2004, Constantinopoulos *et al.* 2006, White *et al.* 2014). A variety of approaches are used to optimize these parameters, including using information criterion such as BIC or ICL within an EM framework (Andrews and McNicholas 2013, Marbac and Sedki 2016) or via a greedy search algorithm over different subsets of variables (Dean and Raftery 2009, Maugis *et al.* 2009), which can be computationally costly due to the wide search space for variable subsets. This motivates methods which perform variable selection simultaneously with cluster allocation. The R package PReMiuM includes simultaneous inference for either binary or continuous latent covariate selection variables in an MCMC framework, though at a high computational cost. For a comprehensive review of variable selection methods in model-based clustering, see e.g. Fop and Murphy (2018).

The availability of diverse ’omics datasets not only motivate the need for methods applicable to high-dimensional problems, but also motivates the need for methodology for integrative analysis. The improvement in performance of statistical analysis of these datasets when considering a diverse range of ’omics data together has been widely explored in the literature (Kirk *et al.* 2012). The Cancer Genome Atlas (TCGA) consortium have defined an abundance of cancer subtypes by integrating ’omics data including DNA methylation, gene expression, micro-RNA sequencing and somatic copy number data (The Cancer Genome Atlas Research Network 2011, The Cancer Genome Atlas Network 2012). We focus on applications to categorical datasets, including binary (2-category) data, where the lack of ordinal structure presents a challenge in comparison to continuous data, which is more widely researched. Examples of relevant ’omics datasets include indicating the presence of any genetic somatic mutation, or datasets studying copy number aberrations in cancer (amplification/deletion/neither).

In the next section, we present VICatMix, a Bayesian finite mixture model allowing for variable selection via simultaneous optimization of latent variable selection indicators. We utilize variational learning for computational efficiency while also incorporating ideas from Bayesian model averaging. These approaches aim to improve inference for the true number of clusters and provide a more stable characterization. In Sections 3 and 4, we present examples and results on both simulated and real-world biological data, including an integrative clustering example. By applying VICatMix to real-world datasets, we are able to assess the biological relevance of clusters using current scientific knowledge, and compare the alignment of our clusters with existing analyses. Finally, in Section 5, we discuss the results and future work.

## 2 Methods

### 2.1 Finite mixture models with variable selection

In a finite mixture model, we model the generating distribution for the data as a mixture of a finite number K of underlying distributions, allowing us to identify distinct subgroups of similar objects. Each observation is generated by one of the K components, with its underlying distribution capturing the characteristics of the cluster. The general equation is given by:

(1) $$P(X|\pi ,\Phi )=\sum_{k=1}^{K}\pi_{k}f(X|\Phi_{k}),$$

where the component densities $f(X|\Phi_{k})$ are from the same parametric family but with different parameters associated with each component. In our case, we model each component with a categorical distribution for each of the P random variables (which is equivalent to the Bernoulli distribution if there are two categories). The model parameters are given by $\Phi_{kj}=[\phi_{kj1},\ldots ,\phi_{kjL_{j}}]$, where $\phi_{\mathrm{kjl}}$ represents the probability of variable j taking value l on component k, and $L_{j}$ is the number of categories for variable j.

In Equation (1), $X=\{x_{1},\ldots ,x_{N}\}$ denotes our observed data, where $x_{n}$ represents one observation of P categorical random variables. We denote the mixture weight associated with the k-th component by $\pi_{k}$, which satisfy $\sum_{k=1}^{K}\pi_{k}=1$ and $\pi_{k}\geq 0$, and denote the parameters associated with the k-th component by $\Phi_{k}={\left\{\Phi_{kj}\right\}}_{j=1,\ldots ,P}$.

#### 2.1.1 Variable selection

As in Law *et al.* (2004) and Tadesse *et al.* (2005), we introduce binary variable selection indicators $\gamma_{j}$ such that $\gamma_{j}=1$ if and only if the j-th covariate is selected for inclusion in the mixture model. We now write for the probability density for a data point $x_{n}$ in a cluster k:

(2) $$f(x_{n}|\Phi_{k})=\prod_{j=1}^{P}f_{j}{\left(x_{nj}|\Phi_{kj}\right)}^{\gamma_{j}}f_{j}{\left(x_{nj}|\Phi_{0j}\right)}^{1-\gamma_{j}}$$

 $\Phi_{0j}=[\phi_{0j1},\ldots ,\phi_{0jL_{j}}]$ represents parameter estimates obtained for covariate j under the null assumption that there exists no clustering structure in the j-th covariate.

### 2.2 Variational Bayesian mixture models

To complete the model specification for our Bayesian finite mixture model, we introduce priors for the model’s parameters as follows:

(3) $$\pi =(\pi_{1},\ldots ,\pi_{K})∼\text{Dirichlet}(\alpha_{0}),$$

 

(4) $$\phi_{kj}=(\phi_{kj1},\ldots ,\phi_{kjL_{j}})∼\text{Dirichlet}(\epsilon_{j}),$$

 

(5) $$\gamma_{j}|\delta_{j}∼\text{Bernoulli}(\delta_{j}),$$

 

(6) $$\delta_{j}∼\text{Beta}(a,a),$$

where all Dirichlet priors are symmetric in our set-up. We set K to be higher than the number of clusters we expect to find, and set $\alpha_{0}<1$. It has been shown theoretically that if $K>K_{\text{true}}$ (the true number of clusters), then—under certain assumptions—setting $\alpha_{0}<1$ in the symmetric Dirichlet prior for the mixture weight allows the true posterior to asymptotically converge to $K_{\text{true}}$ clusters as the number of observations goes to infinity, where superfluous components are emptied and the true number of clusters can be determined by counting the number of non-empty clusters (Rousseau and Mengersen 2011, Malsiner-Walli *et al.* 2014, van Havre *et al.* 2015). This is known as an overfitted mixture model, or a sparse finite mixture, where the sparse Dirichlet prior favours mixture weights close to 0 (Frühwirth-Schnatter and Malsiner-Walli 2018).

We set $\epsilon_{j}=1/L_{j}$ for each j=1,…,P, representing no prior favouring for a variable to take any certain value within any cluster.

The hyperprior for δ, the hyperparameter for the Bernoulli prior on γ, allows the prior probability of including each covariate to be a target for inference in the model. We see in experiments (Supplementary Fig. S6) that the model’s accuracy is robust to the choice of a, so we set a=2 in all studies. When fitting the model, we set $c_{j}=E_{\gamma }(\gamma_{j})=1$ initially for all j=1,…,P, so all variables are initially included and the model gradually removes variables if it does not support its inclusion.

#### 2.2.1 Variational inference

We adopt a variational approach to inference, approximating the posterior parameter distribution by a distribution q(θ) that maximizes the Evidence Lower Bound (ELBO), which is equivalent to minimizing the Kullback-Leibler divergence between p(θ|X) and q(θ). We constrain q to be a mean-field approximation, so it is a product of the form $q(\theta )=q_{Z}(Z)q_{\pi }(\pi )q_{\Phi }(\Phi )q_{\gamma }(\gamma )q_{\delta }(\delta )$, and Z is a collection of latent variables for the data points representing their cluster allocations (see Supplementary Material).

The variational update equations are provided in the Supplementary Material, where, to accelerate computation, we precompute estimates of the parameters $\Phi_{0j}$ associated with the covariates that do not contribute to the definition of the clustering structure, as in Savage *et al.* (2013).

**Figure 1.**
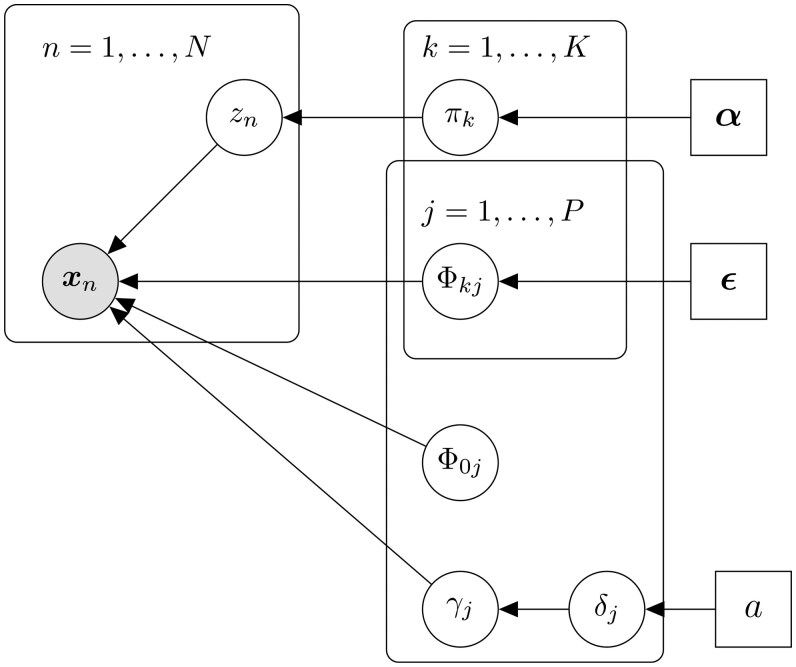
Graphical representation of VICatMix. Here, $z_{n}$ is a ‘1-of-K’ latent variable associated with the data point $x_{n}$ representing its cluster allocation; see the Supplementary Material for more details.

Figure 1 provides a graphical representation of our variational Bayesian mixture model with variable selection.

### 2.3 Summarizing and Bayesian model averaging

Since the ELBO is a non-convex objective function, VI procedures can only guarantee convergence to a local optimum, which can be sensitive to initialization (Bishop 2006, Altosaar *et al.* 2017). A standard approach to addressing this challenge is to perform multiple runs of VICatMix with different random initializations, and then to select from among these the run that provides the maximum ELBO (Ueda and Ghahramani 2002, Friston *et al.* 2007). Here we use ideas from post-processing of MCMC clustering algorithms and the SUGSVarSel approach of Crook *et al.* (2019) to ‘average’ out over a range of initializations to generate a single summary clustering $Z^{*}$. This averaged model is referred to as VICatMix-Avg. In other contexts, we have found that such approaches can help to reduce the identification of spurious singleton clusters (Chaumeny *et al.* 2022).

The central quantity we use to process and summarize *M* clustering solutions, labelled m=1,…,M, is a NxN co-clustering matrix *P*. This matrix summarizes the consistency of cluster assignments across different runs of the algorithm by capturing the frequency in which pairs of data points are assigned to the same cluster, and is defined by:

(7) $$P_{ij}=p(z_{i}=z_{j}|X)\approx \frac{1}{M}\sum_{m=1}^{M}I[z_{i}^{\left(m\right)}=z_{j}^{\left(m\right)}],\;i,j=1,\ldots ,N$$

where I represents the indicator function, and $z_{i}^{\left(m\right)}$ is the latent variable representing the cluster allocation for observation i in clustering solution m. Each entry of the matrix represents an estimation of the probability that two observations will appear in the same cluster, and is analogous to the posterior similarity matrix (Fritsch and Ickstadt 2009) used in post-processing of MCMC samples. We then use this quantity to find a summary representative clustering $Z^{*}$. Having evaluated the co-clustering matrix, we consider two previously proposed methods for identifying a final summary clustering: ‘Medvedovic’ clustering, where 1−P is a distance matrix for agglomerative hierarchical clustering (Medvedovic *et al.* 2004), and variation of information (VoI) (Meilă 2007, Wade and Ghahramani 2018) with optimization methods ‘average’ and ‘complete’. See the Supplementary Material for more details on these methods and their implementation.

#### 2.3.1 Summarizing the selected variables

We summarize the variables selected over M runs (each with a different random initialization), by first calculating the proportion of runs in which each variable was selected, and then thresholding these proportions to identify a final summary set of selected variables. We consider thresholds τ=0.5 and 0.95—see Supplementary Information for full details.

### 2.4 Implementation and availability

The model, VICatMix is freely available as an R package via CRAN, incorporating C++ for faster computation, at https://CRAN.R-project.org/package=VICatMix

##  

### 2.5 Simulation setup

Examples in this section use simulated binary data; an example simulation study using categorical data with 3 categories can be found in Supplementary Section S5.6.

#### 2.5.1 Individual VICatMix simulations

We generate sample binary data with N observations with P independent covariates. Observations are assigned to clusters in given proportions for each cluster in the dataset. For observations in a certain cluster, the probability P of each covariate taking the value ‘1’ is randomly generated via a Beta(1,5) distribution. Different values of P are generated for every cluster and every covariate, encouraging sparse probabilities varying across clusters. For noisy variables, the probability of a ‘1’ is also generated by a Beta(1,5) distribution but this probability is the same regardless of the observation’s cluster membership.

First, we assess the correlation between (log)-ELBO and the quality of the clustering solution. We also test the effects of changing the α parameter to control prior sparsity in our model between a range of values {0.005, 0.01, 0.05, 0.1, 0.5, 1, 5} above and below 1, which was the threshold for an overfitted mixture model as in Section 2.2. We look at the effects of varying the initial number of clusters, $K_{\text{init}}$, on the resulting number of non-empty clusters and accuracy of the clustering. Finally, we look at varying the a hyperparameter. Further details and results are in the Supplementary Material.

#### 2.5.2 VICatMix-Avg simulations

In our second simulation study, we demonstrate the improvement in accuracy for VICatMix when we implement model averaging as described previously. We generate 10 independent datasets and run VICatMix or VICatMixVarSel with 30 different initializations on each dataset. We compare the effects of using Medvedovic clustering and VoI with average and complete linkage as summarization methods when using 5, 10, 15, 20, 25, and 30 different clustering solutions in the co-clustering matrix, and refer to the averaged models as VICatMix-Avg or VICatMixVarSel-Avg. An outline of the different simulation scenarios for this study is given in Table 1. Additionally, we look again at the effects of varying the initial number of clusters, $K_{\text{init}}$ as in Section 3.1.1, but now using VICatMix-Avg.

**Table 1.** Table giving parameters for data generation for the second and third simulation studies.

ID	% relevant variables	N	P	$K_{\text{init}}$	$K_{\text{true}}$	N per cluster
2.1	100%	1000	100	30	10	50–200
2.2	100%	1000	100	30	10	10–400
2.3	100%	2000	100	40	20	50–200
2.4	75%	1000	100	30	10	50–200
2.5	50%	1000	100	30	10	50–200
3.1	100%	1000	100	20	10	100
3.2	100%	1000	100	20	10	25–400
3.3	100%	2000	100	30	20	50–200
3.4	75%	1000	100	20	10	50–200
3.5	50%	2000	100	20	10	100–400

Simulations 2.1, 2.2, and 2.3 were run without variable selection; Simulations 2.4, 2.5 were run with variable selection. $K_{\text{init}}$ refers to the initialized value of *K* in VICatMix.

#### 2.5.3 Comparator methods

We compare to the following existing methods: PReMiuM (Liverani *et al.* 2015), Bayesian Hierarchical Clustering (BHC) (Heller and Ghahramani 2005, Savage *et al.* 2009), BayesBinMix (Papastamoulis and Rattray 2017), FlexMix (Leisch 2004) and agglomerative hierarchical clustering (Kaufman and Rousseeuw 1990, Langfelder and Horvath 2012). Further details for the settings and implementation for them is provided in the Supplementary Material. An outline of our five simulation scenarios is captured in Table 1. All models are compared to VICatMix in Simulations 3.1, 3.2, and 3.3 without variable selection; in Simulations 3.4 and 3.5, we compare VICatMixVarSel with VICatMix (without variable selection) as well as with PReMiuM, which incorporates variable selection, and BHC as a comparison to a method with no variable selection to see if its performance diminishes with noisier data.

#### 2.5.4 Run-times

As the main advantage of using VI as opposed to MCMC is its computational efficiency, our last simulation study measures the time taken to run the model. We first run the model for fixed P=100 and N∈{100,200,500,1000,2000,5000,10 000,20 000,50 000}, and then fixed N=1000 and P∈{10,20,50,100,200,500,1000,2000}. In all cases, we run VICatMix (without averaging) on 10 independently generated datasets with 10 true clusters each, and initialize with K=20. In the variable selection case, 80% of the variables are relevant.

#### 2.5.5 Evaluation of results

Across our simulations, we use the adjusted Rand Index (ARI) (Rand 1971, Hubert and Arabie 1985) to assess the quality of our clustering solutions compared to the true simulated labels, where 1 denotes an exact match to the true labels, and 0 represents a clustering solution which is no better than random allocation. We look at comparisons of the number of clusters detected and run-times in all model comparison simulations. We also use $F_{1}$ scores to quantify the quality of variable selection in each variable selection simulation, which represents a harmonic mean between precision and recall (Sundheim 1992).

### 2.6 Yeast galactose data analysis

We consider a 205-gene subset of a dataset by Ideker *et al.* (2001) commonly used for the validation of clustering methods, which consists of discretized gene expression data from experiments representing systematic perturbations of the yeast galactose-utilization pathway.

DNA microarrays were used to measure mRNA concentrations under 20 different experimental conditions in yeast in the presence/absence of galactose and raffinose, and the experiment was replicated four times. Each column of the dataset represents mean results for one experimental condition and the rows are a subset of 205 yeast genes (Yeung *et al.* 2003) used in other studies in the literature (Medvedovic *et al.* 2004, Qin 2006, Fritsch and Ickstadt 2009).

The gene expression data has been discretized into 3 categories representing under-, over- or unchanged expression as in Savage *et al.* (2010), allowing the analysis to be more robust to non-Gaussian noise common in gene expression data, a popular approach in the literature [eg. (Gerber *et al.* 2007)]. More information about this pre-processing can be found in Savage *et al.* (2010), and the code we used to replicate this categorization is available. We run VICatMix on this 3-category dataset, and as in previous analyses, we compare our results to the classification defined by four Gene Ontology (GO) functional categories (Ashburner *et al.* 2000).

### 2.7 Acute myeloid leukaemia data analysis

We demonstrate the performance of variable selection in VICatMix with an application to Acute Myeloid Leukaemia (AML) mutation data from the TCGA Research Network (Cancer Genome Atlas Research Network *et al.* 2013, Hoadley *et al.* 2018). Mutation data was retrieved via cBioPortal for Cancer Genomics (Cerami *et al.* 2012, Gao *et al.* 2013, de Bruijn *et al.* 2023). Only somatic mutations seen in at least two patients were considered for analysis, as in Zainul Abidin and Westhead (2016); any mutation seen in just one patient does not provide information relevant to clustering, since genes mutated in a single patient offer no basis for comparison with the rest of the patient sample. This left 151 mutated genes and 185 patients in the dataset. We apply VICatMixVarSel-Avg to this binary dataset, where entry (i,j)=1 if patient i has a somatic mutation in gene j.

### 2.8 Pan-cancer cluster-of-clusters analysis

We apply VICatMix to a pre-processed pan-cancer dataset comprising 3527 samples from 12 cancer types studied by TCGA Research Network in a multiplatform integrative analysis (Hoadley *et al.* 2014). In the original analysis, *Hoadley et al.* identified integrated tumour subtypes, which were correlated with but independent of tissue type, and found to hold prognostic value.

The authors produced 5 different clustering structures of the samples based on DNA somatic copy number, DNA methylation, mRNA expression, microRNA expression, and protein expression. These sets of clusters were then integrated to produce a final dataset for clustering, a ‘Matrix of Clusters’, using Cluster-of-Clusters Analysis (COCA). COCA is an integrative clustering method first introduced by TCGA in a study to define subclasses of human breast tumours (The Cancer Genome Atlas Network 2012), and used widely in other integrative cancer ’omics studies (Chen *et al.* 2016, Aure *et al.* 2017).

In COCA, a ‘Matrix of Clusters’ combines different clustering structures from multiple datasets, and this matrix is then clustered to obtain one unified clustering result (Cabassi and Kirk 2020). Given M datasets, for each dataset m=1,…,M, a clustering structure $c^{m}$ is produced via some clustering method with $K^{m}$ clusters in each structure. $K^{m}$ need not be the same for all m. We define $K=\sum_{m=1}^{M}K^{m}$ to be the total number of clusters. The ‘Matrix of Clusters’ is a KxN dimensional binary matrix, where N is the number of observations seen in all datasets, defined as (Cabassi and Kirk 2020):

(8) $${\text{MOC}}_{k,m_{k}}=\{\begin{array}{cc} 1 & \text{for}\;c_{n}^{m}=m_{k},\\ 0 & \text{otherwise}, \end{array}$$

where $c_{n}^{m}$ refers to the cluster assignment for observation n in clustering $c^{m}$, and $m_{k}$ refers to the kth cluster in dataset m. *Hoadley et al.* and other studies (Chen *et al.* 2016, Aure *et al.* 2017) used consensus clustering (Monti 2003) in order to cluster the Matrix of Clusters.

Further details for how the Matrix of Clusters was created, including the clustering method implemented for each individual molecular dataset, can be found in Hoadley *et al.* (2014). Using the same clusters as *Hoadley et al.* for the 5 datasets, we constructed an identical Matrix of Clusters. The code we used to replicate this is available. We apply VICatMix-Avg to the binary Matrix of Clusters to demonstrate the use of VICatMix in integrative clustering.

## 3 Results

### 3.1 Simulated data

Results and analysis from the first, third and fourth simulation studies, including wall-clock run-times for VICatMix and other models, can be found in the Supplementary Material.

#### 3.1.1 VICatMix-Avg simulations

**Figure 2.**
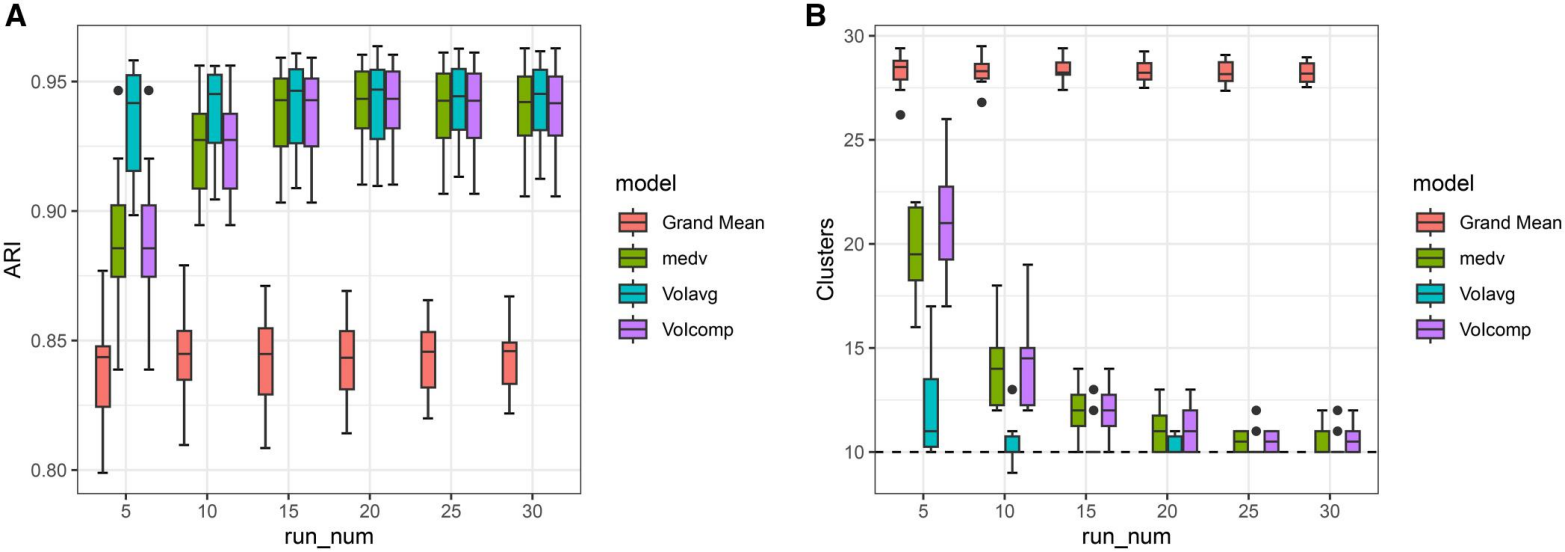
Boxplots comparing the ARI and number of clusters of each model-averaging method across all 10 simulated datasets with the grand mean of the individual runs considered with different numbers of clustering solutions in the co-clustering matrix for Simulation 2.1.

In Simulations 2.1–2.4 (Fig. 2, Supplementary Figs S7–S9, Supplementary Tables S2–S4), all summarization methods showed a significant improvement in ARI compared to each individual run of VICatMix, even with as few as 5 runs in the co-clustering matrix. The number of non-empty clusters was much closer to $K_{\text{true}}$, suggesting that model averaging could mitigate the effect of spurious small clusters. Increasing the number of runs generally improved results. In Simulation 2.4, it is clear that variable selection worked well for clustering of noisier data and model averaging methods further improved accuracy.

Simulation 2.5 (Supplementary Fig. S10) showed that VICatMix became less accurate when more noisy variables are added. It is unsurprising that that averaging over poorer clustering solutions did not lead to the same substantial improvement in accuracy we saw previously—particularly, VoI with average linkage tended to underestimate the true number of clusters as the number of runs was increased, and the median ARI was decreased compared to the median grand mean ARI of the individual runs in all cases. However, we saw an improvement in median ARI for Medvedovic clustering and VoI with complete linkage, and they still corrected for overestimation in the number of clusters with at least 10 runs.

In Simulations 2.4 and 2.5, we looked at which variables were selected using the thresholds detailed in Section 2.3, and illustrate this in Supplementary Figs S11 and S12. Table 2 gives mean $F_{1}$ scores for thresholds τ=0.5 and τ=0.95 across all datasets, showing the implementation of variable selection identified the correct relevant and irrelevant variables with high accuracy. τ=0.95 saw particularly good results in finding the correct irrelevant variables in both simulations.

**Table 2.** Table comparing mean $F_{1}$ scores for variable selection methods under both Simulation 2.4 and Simulation 2.5.

	Simulation 2.4		Simulation 2.5	
Methods	25 Runs	30 Runs	25 Runs	30 Runs
0.5	0.904	0.905	0.750	0.747
0.95	**0.937**	**0.935**	**0.841**	**0.836**

The highest score is shown in bold text.

#### 3.1.2 Implementation recommendation

Paired Wilcoxon rank-sum tests with Bonferroni corrections concluded that there was no statistically significant difference in model accuracy between clustering solutions with at least 25 runs in the co-clustering matrix in all simulations. For example, all pairwise p-values between models with 20–30 initializations were 1 in Simulation 2.1. We determined that including at least 25 runs in our co-clustering matrix is usually sufficient, and that variation of information with complete linkage and τ=0.95 for variable selection are optimal for VICatMix-Avg, although users may wish to tailor this to their specific dataset. These are the default values in the R package. Each run of VICatMix can be run in parallel, and calculating the optimal clustering under VoIcomp took around a second in all our simulations.

### 3.2 Yeast galactose data

We ran VICatMix-Avg with K=4, incorporating the prior knowledge that there are 4 GO functional categories for the data. A heatmap visualizing the results is in Supplementary Fig. S24. We saw that VICatMix-Avg only gave 3 clusters, where GO categories 1 and 3 remained almost intact, and the final cluster was a mix of the two smaller functional groups.

As illustrated in Supplementary Fig. S24, despite some differences, these functional groups have indistinguishable expression in the majority of the variables. Due to their extremely small sizes and many shared patterns, it is reasonable that the algorithm grouped these GO categories together, and could suggest some similarities in the functional groups. Refining the algorithm to robustly separate smaller clusters is an aim in future work.

This clustering structure gave us an ARI score of 0.933 with the four functional groups, comparable with ARI scores using models such as BHC and agglomerative hierarchical clustering for the same dataset in the literature (Yeung *et al.* 2003, Savage *et al.* 2009). A high ARI with the GO functional categories indicates strong agreement between our clustering structure and prior biological knowledge.

**Figure 3.**
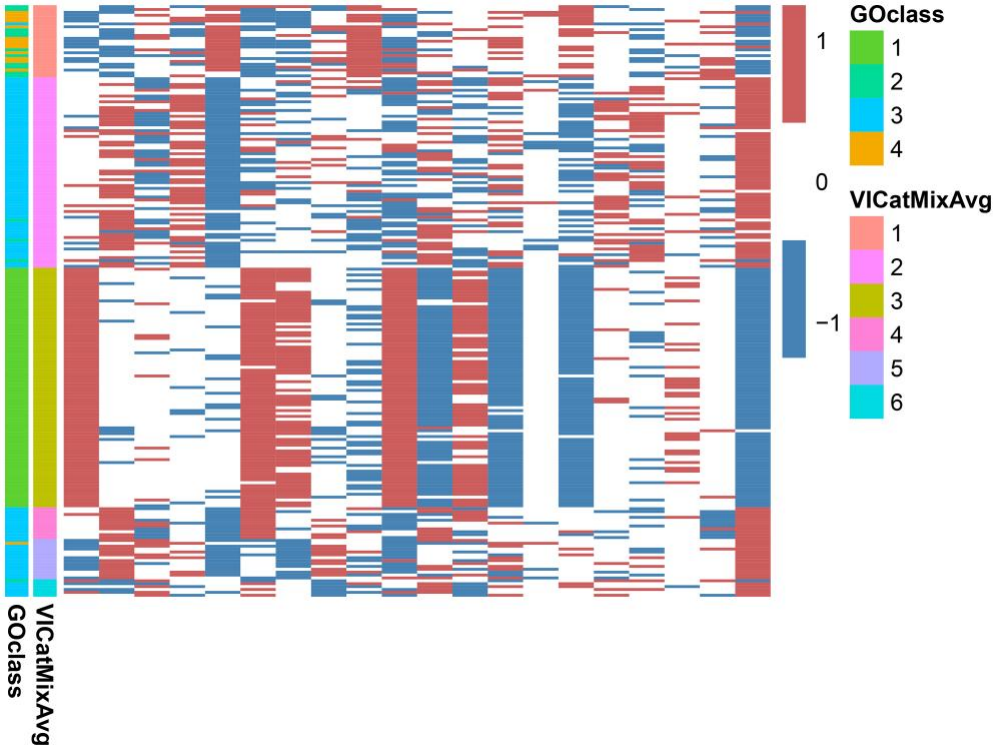
Heatmap of the VICatMix-Avg clustering structure on the yeast galactose data compared with the GO labelling when *K* = 10. ‘−1’, ‘0’, and ‘1’ refer to under-, over- and unchanged expression, respectively.

Motivated by the ability of VICatMix-Avg to automatically detect the true number of clusters in a dataset, we also ran VICatMix-Avg with K=10, which also gave a clearly coherent clustering structure with 6 clusters in Fig. 3. We still saw a cluster which was a mix of the smaller GO functional categories, but GO category 3 was almost perfectly subdivided into three groups, which could infer the existence of biologically significant novel subcategories within the GO functional classes.

### 3.3 Acute myeloid leukaemia data

**Figure 4.**
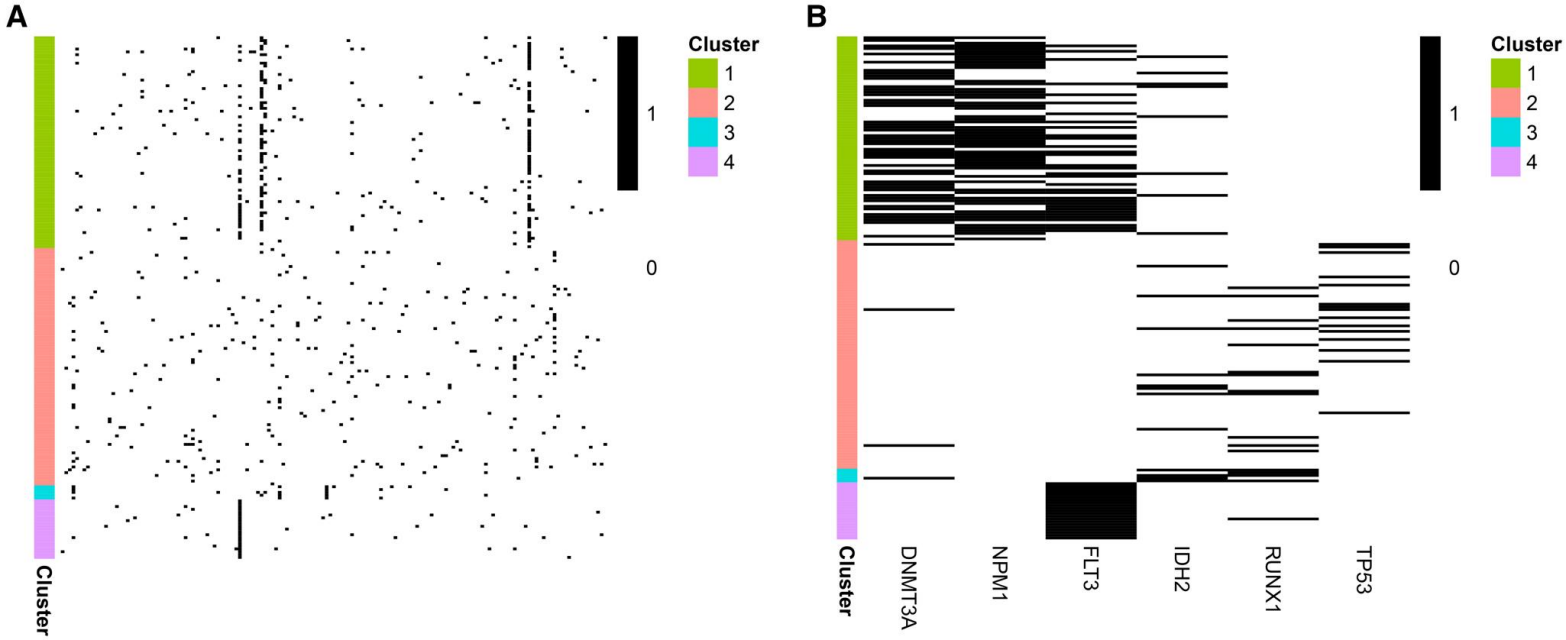
Heatmaps of the VICatMixVarSel-Avg clustering structure on the AML mutation dataset. ‘1’ denotes the presence of a mutation. ‘A’ depicts all variables, ‘B’ shows only the 6 selected variables.

Applying VICatMix without variable selection to the AML dataset led to all samples consistently being put into one cluster. The abundance of noisy variables obscured any clustering structure within the data, demonstrating the need for variable selection methods for the clustering of noisy or sparse data. We therefore applied the VICatMix algorithm with variable selection. Figure 4 depicts heatmaps of the clustering structure generated by VICatMixVarSel-Avg with 30 initializations, where we found 6/151 genes to be selected in more than 95% of the runs. In summary: by applying the VICatMix algorithm with variable selection, a consistent clustering structure across multiple initializations associated with a small subset of the genes could be found, which could not be identified when clustering without variable selection.

**Figure 5.**
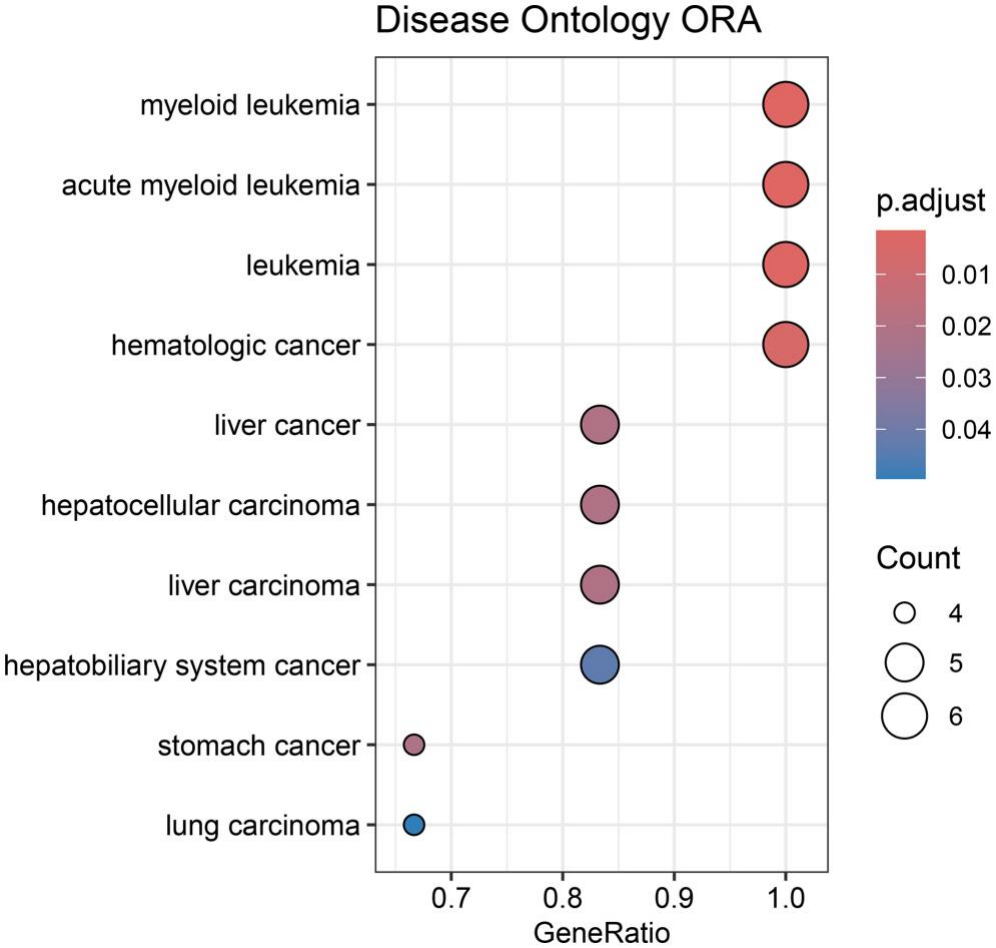
Dotplots visualizing ORA for 6 selected genes for the AML dataset using gene-disease annotations from the DO. *P*.adjust is the *P*-value from the hypergeometric test used in ORA, adjusted using the Benjamini-Hochberg procedure. The top 10 most significant annotations are shown.

We performed overrepresentation analysis (ORA) for the 6 selected genes—DNMT3A, NPM1, FLT3, IDH2, RUNX1, TP53 (Boyle *et al.* 2004) using R/Bioconductor packages ClusterProfiler (Wu *et al.* 2021) and DOSE (Yu *et al.* 2014). ORA uses a hypergeometric test to assess whether certain gene annotations in a repository are statistically enriched or overrepresented in a subset of genes (in our case, the set of selected variables) compared to the full set of genes. When we performed ORA using the Disease Ontology (DO) (Schriml *et al.* 2011) (Fig. 5), we saw that the most significantly overrepresented disease annotations in our gene set were all clearly relevant for AML. DOSE also supports ORA using Network of Cancer Genes (Dressler *et al.* 2022) and DisGeNET (Piñero *et al.* 2019). Supplementary Figure S25 demonstrates that ORA using these repositories further substantiates the association between the 6 selected genes and AML.

Recurrent mutations of all 6 genes have been associated with therapeutic and prognostic implications in AML in the literature and have played a part in molecular classification of AML (DiNardo and Cortes 2016, Shin *et al.* 2016, Yu *et al.* 2020). For example, all 6 genes are noted to be significantly mutated in Shin *et al.* (2016), where a mutation in DNMT3A was shown to be significantly associated with adverse outcome in addition to conventional risk stratification such as the European LeukemiaNet (ELN) classification.

### 3.4 Pan-cancer cluster-of-clusters analysis

**Figure 6.**
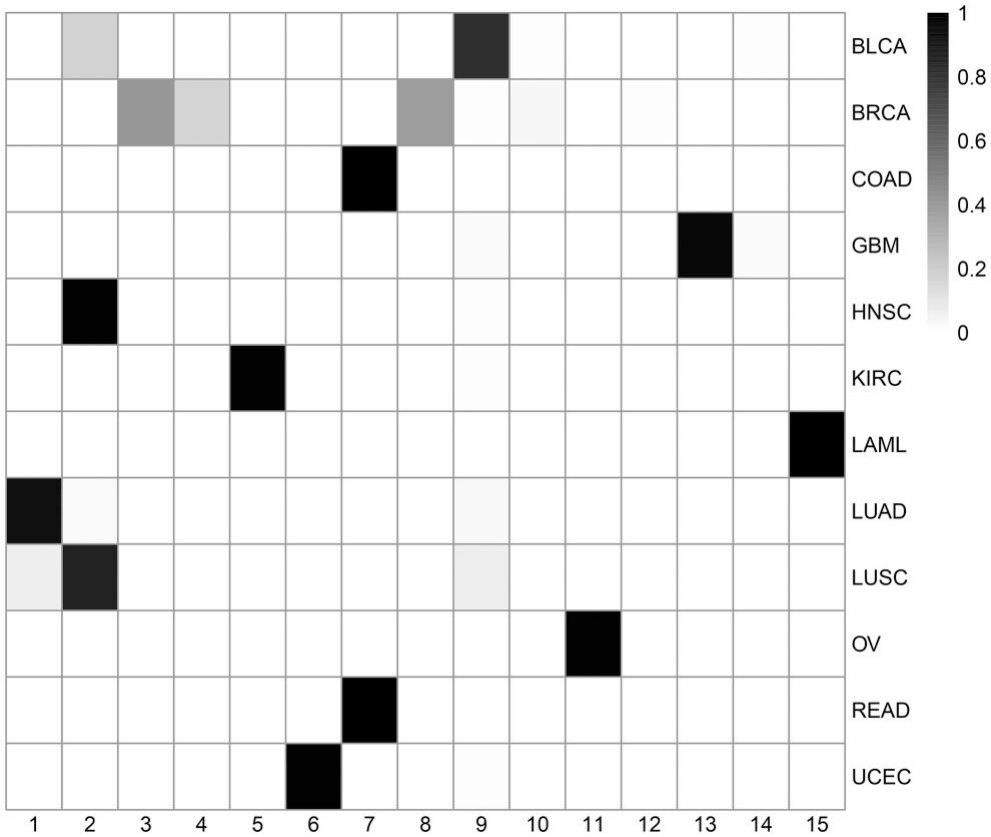
A heatmap showing the correspondence between clusters produced by VICatMix-Avg and the tissues of origin, where K=15. A darker cell colour in row *i* indicates a higher percentage of samples from tissue *i* are in the given cluster *j*.

**Figure 7.**
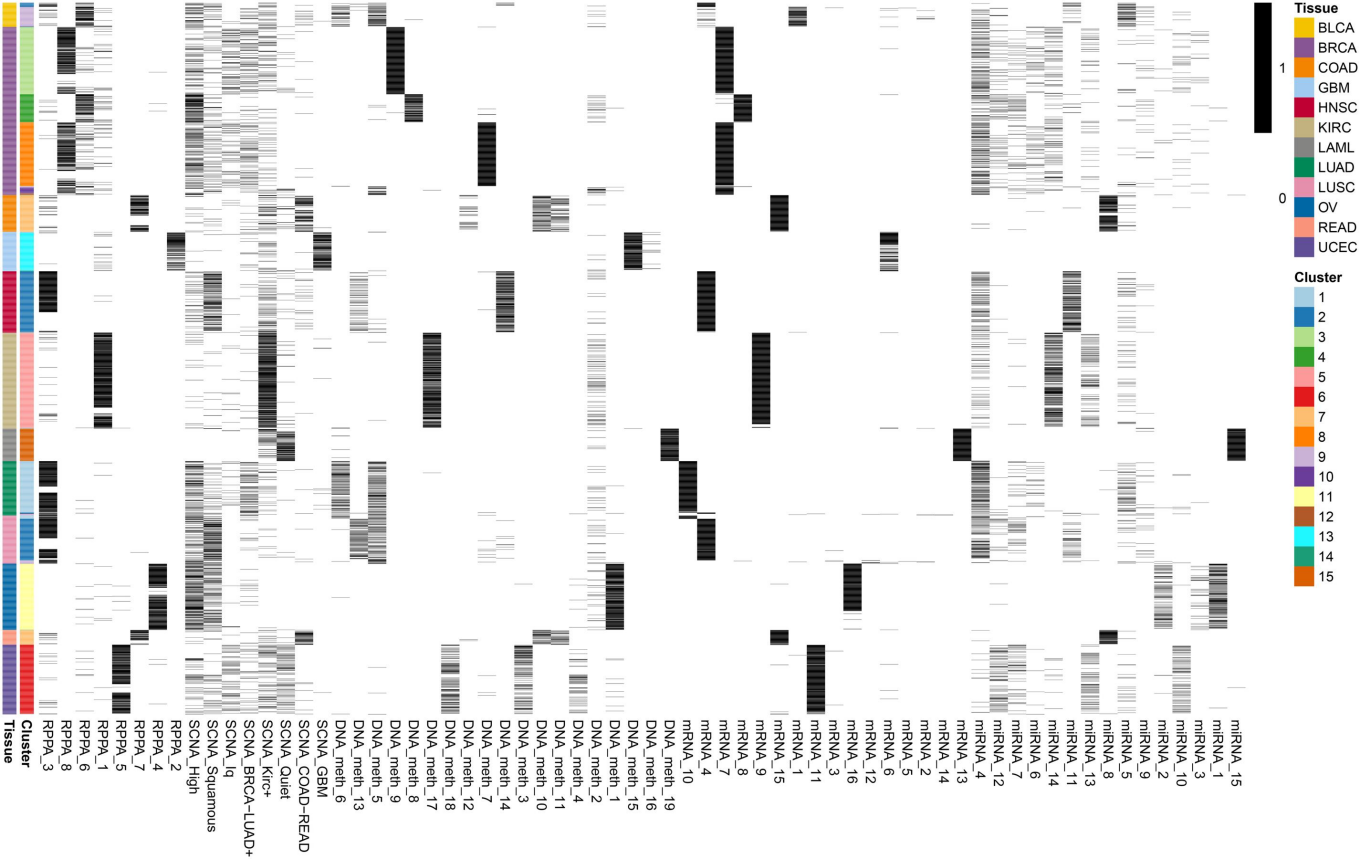
A heatmap depicting the VICatMix-Avg clustering of the Matrix of Clusters for our pan-cancer data, where K=15, and we compare the clusters to the tissue of origin.

We applied VICatMix to our replication of the binary ‘Matrix of Clusters’ that *Hoadley et al.* created. We ran VICatMix-Avg with 25 initializations with no variable selection to incorporate all clustering information. We initially set the upper bound on the number of clusters to be K=15 to enable a similar analysis to the 11 subtypes found by *Hoadley et al.* We found the resulting clusters to correspond to the tissue of origin for the tumour samples as illustrated in Figs 6 and 7. For example, LAML (acute myeloid leukaemia) samples corresponded perfectly with Cluster 15 and Cluster 11 was almost precisely made up of OV (ovarian serous cystadenocarcinoma) samples. We saw a ‘squamous-like’ cluster in Cluster 2 of mostly head and neck squamous cell carcinoma and lung squamous cell carcinoma (LUSC) samples, with other LUSC samples mostly mixing with either lung adenocarcinoma samples in Cluster 1 or BLCA (bladder urothelial carcinoma) samples in Cluster 9, which was similar to analysis by *Hoadley et al.* These results further support the existence of cross-tissue and intra-tissue integrative subtypes as proposed by *Hoadley et al.*

Focusing on the subclusters of BRCA (breast invasive carcinoma) samples, we compared our clusters to PAM50 classificiations (Perou *et al.* 2000, Sørlie *et al.* 2001, Parker *et al.* 2009) by TCGA (Berger *et al.* 2018) (Supplementary Fig. S26). We found that Cluster 4 (which almost completely coincides with the BRCA-Basal cluster of *Hoadley et al.*) was made up of primarily Basal samples, with 132/141 Basal samples falling into this cluster, and this cluster is clearly separated from other BRCA samples.

VICatMix-Avg’s identification of the Basal BRCA subtype, which has been shown to have a statistically significantly different response to chemotherapy and prognostic outlook (Banerjee *et al.* 2006, Parker *et al.* 2009), motivates its application to the identification of other cancer subtypes in integrative ’omics data analysis. To investigate further subclustering structure, corresponding to putative tumour subtypes, we additionally considered K=40. The resulting clusters and a discussion of these results are in Supplementary Section S9.

## 4 Discussion

In this paper, we have presented VICatMix, a variational Bayesian model training a finite mixture model for categorical data including variable selection. VI provides a substantial speed-up where MCMC methods have faltered in the past due to high computational cost.

Our addition of summarization and Bayesian model averaging via a co-clustering matrix mitigates the problems of reaching poor local optima when using VI and allows for improved results. VICatMix-Avg is both faster than many competitors in the literature and also more accurate in terms of the ARI and finding the true number of clusters in simulations.

Applications to real-world data showed that VICatMix was able to identify biologically relevant clusters in line with existing scientific knowledge, and demonstrated its potential use in future exploratory analysis of novel biomedical datasets. For example, VICatMix found clusters aligning with GO functional categories in the case of yeast galactose data, and an application to a TCGA pan-cancer dataset showed that VICatMix was able to identify similar integrated tumour subtypes to that of *Hoadley et al.*, including the identification of a known breast cancer subtype. It would be interesting to follow the entire COCA workflow with our own clustering analysis for each type of ’omics data, and compare the results we get with the new Matrix of Clusters. This would require tailoring VICatMix to be applicable to other data types including continuous data.

The power of variable selection was seen in the application to AML data, where VICatMixVarSel was able to pick out 6 genes which were statistically significantly enriched for AML out of a total of 151. Applying VICatMixVarSel to similar binary mutation datasets for less frequently studied cancer types could enable the future discovery of new driver genes. Example ’omics datasets in this manuscript concern cancer genomics, but it is expected that VICatMix would be applicable to a range of ’omics data in a binarized or categorized format.

## 5 Conclusion

Overall, VICatMix allows us to bypass the hurdle of computationally expensive MCMC models for cluster analysis of categorical data using VI. While our resulting model is still only an approximation to the true posterior, our results on both simulated and real-world data show remarkable results. Further areas for improvement in the model include implementing cluster-specific regression models to investigate the relationship between covariates and a given response variable, incorporating the ELBO to give different weights for different initializations in the co-clustering matrix for summarization, including ‘merge-delete’ moves to improve efficiency (Hughes *et al.* 2015), and extending to a multi-view model where multiple clustering ‘views’ can be modelled in high-dimensional biomedical data.

## Supplementary Material

vbaf055_Supplementary_Data

## Data Availability

The model, VICatMix is freely available as an R package via CRAN, incorporating C++ for faster computation, at https://CRAN.R-project.org/package=VICatMix. The data underlying this article used in real-world simulations are available from online repositories. Citations for the datasets considered are provided in the main body of the text. Code to reproduce all results is available at https://github.com/j-ackierao/VICatMix-paper.
