# Supplementary material for: VICatMix: variational Bayesian clustering and variable selection for discrete biomedical data
Source: Bioinform Adv. 2025 Mar 17;5(1):vbaf055. doi: 10.1093/bioadv/vbaf055 (PMC11981716; doi:10.1093/bioadv/vbaf055)
Supplement: vbaf055_Supplementary_Data [file vbaf055_supplementary_data.pdf]

VICatMix: variational Bayesian clustering and variable selection for discrete biomedical data

Jackie Rao and Paul D. W. Kirk

Supplementary Material

Contents

S1 Mixture models 2

S2 Variational inference 2

S3 Variational updates 3

S4 Summarising and Bayesian model averaging 5
- S4.1 Variation of information 5
- S4.2 Medvedovic clustering 5
- S4.3 Summarising the selected variables 5

S5 Simulated data - results and analysis 7
- S5.1 VICatMix simulations - results 7
  - S5.1.1 Correlation between log-ELBO and adjusted Rand Index (ARI) 7
  - S5.1.2 Effect of varying α 7
  - S5.1.3 Effect of varying initial number of clusters 7
  - S5.1.4 Effect of varying a 8
- S5.2 VICatMix-Avg simulations - additional figures 11
  - S5.2.1 Effect of varying solutions in co-clustering matrix 15
  - S5.2.2 Effect of varying initial number of clusters 15
- S5.3 Comparisons to other models - set-up 17
- S5.4 Comparisons to other models - results and analysis 17
- S5.5 Run-times 19
- S5.6 Categorical simulation study 20

S6 Yeast galactose data 24

S7 Acute myeloid leukaemia (AML) 25

S8 Pan-cancer cluster-of-clusters analysis with K = 15 - additional figures 26

S9 Pan-cancer cluster-of-clusters analysis with K = 40 27

S1 Mixture models

We introduce latent variables z_n associated with each data point x_n which is a '1-of-K' binary vector in $\mathbb{R}^K$ with exactly one non-zero element; $z_{nk} = 1$ if and only if x_n is associated with the k-th component. This allows us to ease computation for the variational algorithm. We now rewrite Equation (1) in the main paper as:

$$p(X|Z, \pi, \Phi) = \prod_{n=1}^{N} \prod_{k=1}^{K} f(\mathbf{x}_n|\Phi_k)^{z_{nk}} \tag{1}$$

We also write down the conditional distribution of the latent variables Z given the mixing coefficients π:

$$p(Z|\pi) = \prod_{n=1}^{N} \prod_{k=1}^{K} \pi_k^{z_{nk}}; \tag{2}$$

The overall conditional distribution for the observed data, given the latent variables and component parameters, in the case of variable selection, is given by:

$$p(X|Z, \pi, \Phi, \gamma) = \prod_{n=1}^{N} \prod_{k=1}^{K} (\prod_{j=1}^{P} \phi_{kjx_{nj}}^{\gamma_j} \phi_{0jx_{nj}}^{1-\gamma_j})^{z_{nk}} \tag{3}$$

S2 Variational inference

In variational inference (VI), we approximate $p(\theta|X)$ with a tractable distribution $q(\theta)$. We have that the following holds for any arbitrary distribution $q(\theta)$:

$$\ln p(X) = \mathcal{L}(q) + KL(q||p), \tag{4}$$

where

$$\mathcal{L}(q) = \int q(\theta) \ln \left(\frac{p(X, \theta)}{q(\theta)} \right) d\theta \tag{5}$$

$$KL(q||p) = - \int q(\theta) \ln \left(\frac{p(\theta|X)}{q(\theta)} \right) d\theta. \tag{6}$$

$KL(q||p)$, the Kullback-Leibler (KL) divergence between $p(\theta|X)$ and $q(\theta)$, is always non-negative, so we see that $\mathcal{L}(q)$, often known as the ELBO (Evidence Lower Bound), is a lower bound for the (log) marginal likelihood, with equality if and only if $p(\theta|X) = q(\theta)$. Therefore, if we maximise $\mathcal{L}(q)$ with respect to $q(\theta)$, we minimise the KL divergence between $p(\theta|X)$ and $q(\theta)$. We can adopt an iterative procedure to optimise $\mathcal{L}(q)$, the ELBO, analogous to the EM-algorithm, and cycle between optimising $q(\theta)$ with respect to each parameter in order for our variational approximation to be as close to the targeted posterior distribution. At each iteration, we can calculate the value of the ELBO function and monitor this value for convergence.

We constrain q to be a mean-field approximation, so it is a product of the form $q(\theta) = q_Z(Z)q_\pi(\pi)q_\Phi(\Phi)q_\gamma(\gamma)q_\delta(\delta)$. By rewriting $\mathcal{L}(q)$ with $q(\theta)$ in this form, the optimal solution $q_j^*(\theta_j)$ for each component of θ, θ_j, satisfies:

$$q_j^*(\theta_j) = \frac{\exp\left(\mathbb{E}_{i \neq j}[\ln p(X, \theta)]\right)}{\int \exp\left(\mathbb{E}_{i \neq j}[\ln p(X, \theta)]\right) d\theta_j} \tag{7}$$

$$\ln q_j^*(\theta_j) = \mathbb{E}_{i \neq j}[\ln p(X, \theta)] + k, \tag{8}$$

where k is an arbitrary constant ensuring that the density integrates to 1.

S3 Variational updates

The overall conditional distribution for the observed data without variable selection can be recovered from Equation (3) by setting $\gamma_j = 1$ for all j. We decompose the full model over all of our observations, latent variables and parameters including their priors as:

$$p(X, Z, \pi, \Phi) = p(X|Z, \Phi)p(Z|\pi)p(\pi)p(\Phi) \tag{9}$$

In this case, the variational update equations for the cluster allocation latent variables, Z, are given by:

$$q^*(Z) = \prod_{n=1}^{N}\prod_{k=1}^{K} r_{nk}^{z_{nk}}, \qquad r_{nk} = \frac{\rho_{nk}}{\sum_{j=1}^{K}\rho_{nj}} \tag{10}$$

$$\ln \rho_{nk} = \mathbb{E}_{\pi}[\ln \pi_k] + \sum_{i=1}^{P} \mathbb{E}_{\Phi}[\ln \phi_{kix_{ni}}] \tag{11}$$

We call r_{nk} the responsibility of the k-th component for the n-th observation, and $\mathbb{E}[z_{nk}] = r_{nk}$; data point n is allocated to the cluster k with the highest responsibility.

The variational update equations for π, ϕ are given by:

$$q^*(\pi) \propto \prod_{k=1}^{K} \pi_k{}^{\sum_{n=1}^{N} r_{nk}+\alpha_k-1} \tag{12}$$

$$\pi = (\pi_1, ..., \pi_K) \sim \text{Dirichlet}(\alpha_1^*, ..., \alpha_K^*) \tag{13}$$

where $\alpha_k^* = \alpha_k + \sum_{n=1}^{N} r_{nk}$ for $k = 1, ..., K$.

$$q^*(\phi_{kj}) \propto \prod_{l=1}^{L_i} \phi_{kjl}^{(\epsilon_j+\tilde{N_{jl}}-1)} \tag{14}$$

$$\phi_{kj} = (\phi_{kj1}, ..., \phi_{kjL_j}) \sim \text{Dirichlet}(\epsilon_{kj1}^*, ..., \epsilon_{kjL_j}^*) \tag{15}$$

where we let $\tilde{N_{jl}} = \sum_{n=1}^{N} \mathbb{I}(x_{nj} = l)r_{nk}$, and $\epsilon_{kjl}^* = \epsilon_j + \tilde{N_{jl}}$ for all $l = 1, ..., L_i$, $k = 1, ..., K$ and $i = 1, ..., P$..

With variable selection, the full model is now given by:

$$p(X, Z, \pi, \Phi, \gamma, \delta) = p(X|Z, \Phi, \gamma)p(Z|\pi)p(\pi)p(\Phi)p(\gamma|\delta)p(\delta) \tag{16}$$

The form of the Z update remains the same as in Equation (10) but we have that ρ_{nk} is defined instead by:

$$\ln \rho_{nk} = \mathbb{E}_{\pi}[\ln \pi_k] + \sum_{j=1}^{P} c_j \mathbb{E}_{\Phi}[\ln \phi_{kjx_{nj}}] + (1 - c_j)(\ln \phi_{0jx_{nj}}) \tag{17}$$

$c_j = \mathbb{E}_{\gamma}(\gamma_j)$, where the expectation is taken over the variational distribution for γ.

The form of the π update remains the same as in Equations (12), (13). The variational update for ϕ also remains in the same form as in Equations (14), (15), but we redefine $\tilde{N_{jl}} = \sum_{n=1}^{N} \mathbb{I}(x_{nj} = l)r_{nk}c_j$.

The updates for our variable selection parameters γ and δ are given as:

$$q^*(\gamma_i) = c_j^{\gamma_j}(1 - c_j)^{1-\gamma_j}, \qquad c_j = \frac{\eta_{1j}}{\eta_{1j} + \eta_{2j}} = \mathbb{E}_{\gamma}(\gamma_j) \tag{18}$$

$$\ln \eta_{1j} = \sum_{n=1}^{N}\sum_{k=1}^{K}(r_{nk}\mathbb{E}_{\Phi}[\ln \phi_{kjx_{nj}}]) + \mathbb{E}_{\delta}[\ln \delta_j] \tag{19}$$

$$\ln \eta_{2j} = \sum_{n=1}^{N}\sum_{k=1}^{K}(r_{nk} \ln \phi_{0jx_{nj}}) + \mathbb{E}_{\delta}[\ln(1 - \delta_j)] \tag{20}$$

$$\gamma_j \sim \text{Bernoulli}(c_j) \tag{21}$$

$$q^*(\delta_j) \propto \delta_j^{(c_j+a-1)}(1-\delta_j)^{(1-c_j+a-1)} \tag{22}$$

$$\delta_i \sim \text{Beta}(c_j + a, 1 - c_j + a) \tag{23}$$

All expectations throughout are taken over the variational distributions for each parameter. The general algorithm now involves cycling between estimating the r_{nk} by using the current variational distributions $q^*(\pi)$, $q^*(\phi)$ and the current value of $c_j = \mathbb{E}_\gamma(\gamma_j)$ (when using variable selection) to calculate the expectations in Equation (17) ('variational E step'), and then using these to recompute the parameters in the variational distributions for $\pi, \Phi, \gamma, \delta$ ('variational M step'). We initialise using k-modes (Chaturvedi, Green, and Caroll, 2001), a method analogous to k-means for categorical data.

S4 Summarising and Bayesian model averaging

S4.1 Variation of information

The most 'representative' clustering $\mathbf{Z}^*$ given a co-clustering matrix is not well defined, but Binder introduced a formal definition under a Bayesian decision theory framework (Binder, 1978). Given a loss function L, the most 'representative' clustering $\mathbf{Z}^*$ should satisfy:

$$\mathbf{Z}^* = \arg\min_{\hat{z}} \mathbb{E}[L(z, \hat{z})|X] \tag{24}$$

One such loss function L which can be used in the framework in Equation 24 is the variation of information (VoI), constructed using information theory and introduced by Meilă (Meilă, 2007) for cluster comparison between clustering solutions c and c'. Intuitively, this equation compares the information captured in each clustering individually with the information that is shared between both clusterings; the VoI is therefore small when the information shared by both clustering structures is close to the sum of the individual information captured by each clustering.

This was first proposed as a loss function by Wade and Gharamani (Wade and Ghahramani, 2018), who found that VoI has many desirable theoretical properties in the distance space in order to be used as a loss function, and also found a computationally efficient lower bound on the expected loss only depending on the posterior through the co-clustering matrix. We implement VoI via the function *minVI* in the R package *mcclust.ext* and consider the optimisation methods 'average' and 'complete', where the search space is restricted to clusterings found via hierarchical clustering (with distance metric $1-P$) with either average or complete linkage; computing the lower bound for every possible clustering in the search space is infeasible.

VoI has been found to be successful in finding accurate summary clusterings and correct for the overestimation in the number of clusters in many Markov Chain Monte Carlo (MCMC) simulations (Chaumeny et al., 2022; Wade and Ghahramani, 2018; Rastelli and Friel, 2017; Lijoi, Prünster, and Rebaudo, 2022), especially compared to other popular loss functions such as Binder's loss (Binder, 1978) and the posterior expected adjusted Rand index (Fritsch and Ickstadt, 2009).

S4.2 Medvedovic clustering

An alternative method to find $\mathbf{Z}^*$ is what we refer to as Medvedovic clustering (Medvedovic, Yeung, and Bumgarner, 2004), where $1 - \mathbf{P}$ is used as a distance matrix for agglomerative hierarchical clustering with complete linkage. Complete linkage allows the number of clusters to be determined by cutting the tree at a certain linkage distance $1 - \epsilon$ (Fritsch and Ickstadt, 2009); Fritsch and Ickstadt use a value of $\epsilon = 0.01$.

This approach has been criticised as being *ad hoc* with little theoretic basis; it cannot be expressed in the decision theory framework in Equation (24), and there is no clear principle to choose where to cut the tree, although Fritsch and Ickstadt proved that using $1 - P_{ij}$ as the distance between two observations does hold certain desirable properties for a distance measure as a topological pseudometric for the space of observations (Fritsch and Ickstadt, 2009). Nevertheless, Medvedovic clustering has shown good results across many biological scenarios (Crook, Gatto, and Kirk, 2019; Rasmussen et al., 2009). Medvedovic clustering is implemented in the R package *mcclust* in the function *medv*.

S4.3 Summarising the selected variables

To identify a final summary set of selected variables, we consider a threshold τ for the proportion of runs in which a variable is selected in. Given M runs with different initialisations, a variable j is selected if:

$$\frac{\sum_{m=1}^{M} \mathbb{1}(c_j^{(m)} = 1)}{M} > \tau, \tag{25}$$

where $c_j^{(m)}$ is the value of $c_j = \mathbb{E}(\gamma_j)$ in run m. We found that in many circumstances, c_j fails to converge exactly to 1 but usually gets extremely close to 1, so in practice, we take variable j to be selected if:

$$\frac{\sum_{m=1}^{M} \mathbb{1}(c_j^{(m)} > 0.5)}{M} > \tau, \tag{26}$$

S5 Simulated data - results and analysis

S5.1 VICatMix simulations - results

S5.1.1 Correlation between log-ELBO and adjusted Rand Index (ARI)

In Figure S1, we look at the correlation between log-ELBO and adjusted Rand Index (ARI) across 5 different batches of simulated data, and run VICatMix 20 times on each batch of simulated data. There was a strong positive correlation between log-ELBO and ARI (with p-values < 0.001 in all cases (Table S1), justifying picking the clustering with the highest (log-)ELBO as the 'optimal' run given many clustering structures with different intialisations. Individual runs of VICatMix performed very well, with an ARI more than 0.80 in almost every initialisation on every dataset.

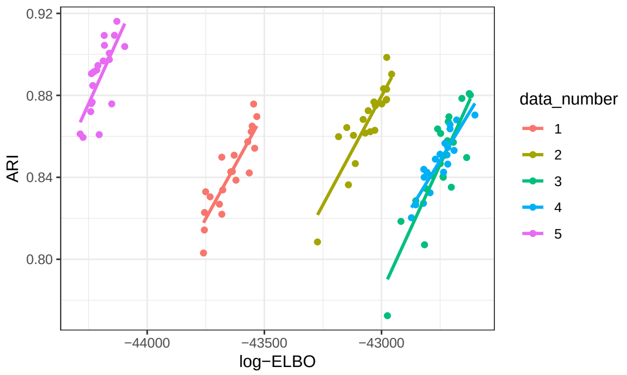

Figure S1: Graph showing the correlation between log-ELBO and ARI across different initialisations of VICatMix on 5 different sets of simulated data.

Table S1: Table giving the Pearson correlation coefficient and the p-value of the correlation for each dataset.

Data Number	Correlation Coeff.	p-value
1	0.880	0.00000031
2	0.884	0.00000024
3	0.861	0.0000011
4	0.896	0.000000091
5	0.731	0.00025

S5.1.2 Effect of varying α

We then looked at varying α, where we simulated datasets with $N = 1000$, $P = 100$, 10 evenly sized true clusters initialised with $K = 30$, and look at 10 different initialisations of the model. We also tested this with the same data dimensions, but with 4 unevenly sized true clusters and $K = 10$. In Figure S2, we saw that when we varied α, the number of clusters in the final converged model still remained close to the initialised value of K, despite using an overfitted mixture model. Even if the true posterior behaviour leads to the emptying of negligible clusters, our model only gave us a local optimum where observations were still stuck in small clusters of less than 5 observations. This motivates the use of summarisation and Bayesian model averaging as described in Section 2.3 of the main paper. Figure S3 shows that varying α did not have an obvious effect on the ARI.

S5.1.3 Effect of varying initial number of clusters

Looking at varying the initial number of clusters, K_{init}, we simulated 10 datasets with $N = 1000$, $P = 100$ and 4 true clusters, then 10 datasets with $N = 1000$, $P = 100$ and 10 true clusters, and

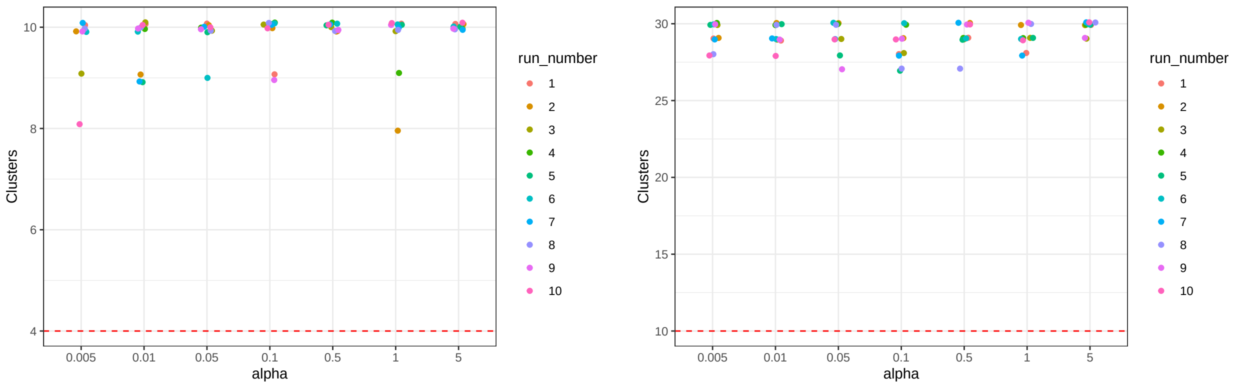

(a) 4 true clusters, K=10

(b) 10 true clusters, K=30

Figure S2: Two examples of simulated data with $N = 1000$, $P = 100$ initialised 10 times for each value of $\alpha \in \{0.005, 0.01, 0.05, 0.1, 0.5, 1, 5\}$ with the resulting number of clusters plotted and the 'true' number of clusters indicated with a red dashed line. We see that clusters are very rarely emptied.

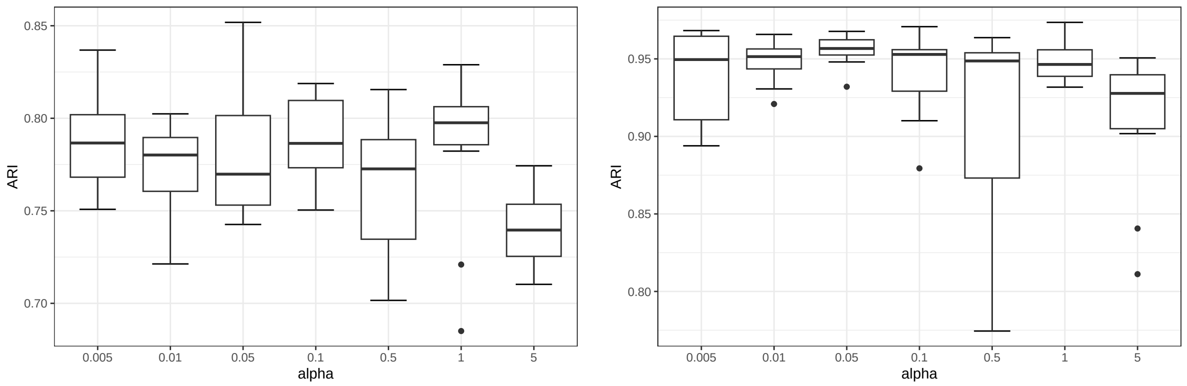

(a) 4 true clusters, K=10

(b) 10 true clusters, K=30

Figure S3: Two examples of simulated data with $N = 1000$, $P = 100$ initialised 10 times for each value of $\alpha \in \{0.005, 0.01, 0.05, 0.1, 0.5, 1, 5\}$ with the resulting ARI plotted.

ran VICatMix 10 times on each of these datasets. In the first scenario, we looked at all numbers of initial clusters between 3 and 10, and in the second scenario, we considered a wider range of initial clusters ($K_{\text{init}} \in \{5, 10, 15, 20, 25, 30\}$). Illustration of our results is seen in Figures S4 and S5. We saw in both scenarios that the resulting number of clusters was either the same as the number of initial clusters, or close to that (including many spurious small clusters), motivating further our use of model averaging and summarisation methods in later sections of the manuscript. ARI values were slightly higher when we considered an initial number of clusters higher than the true number of clusters. This could be explained by the model being more free to explore the optimisation surface with a higher number of initial clusters.

S5.1.4 Effect of varying a

Figure S6 demonstrates the effects of changing the hyperparameter in the hyperprior for δ, where a represents the parameter in the Beta distribution. Visually, there appeared to be no difference in the accuracy of the model when we changed this hyperparameter, and a Kruskal-Wallis test (a non-parametric test to compare the means of the groups) gave a p-value of 0.1598, suggesting that there was no significant difference in the distributions of the groups. We use $a = 2$ as a default throughout the experiments in the report.

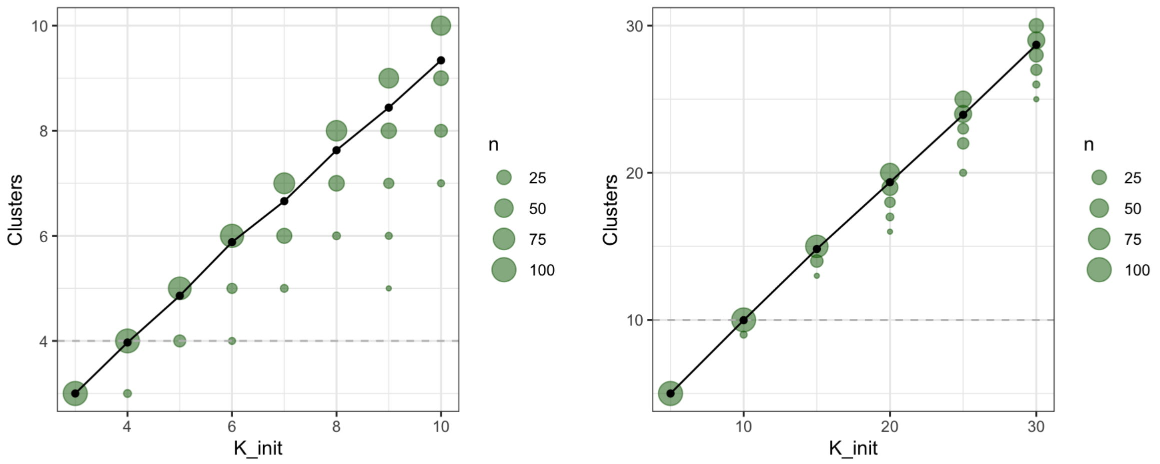

(a) 4 true clusters (sized between 100-400 points)

(b) 10 true clusters (evenly sized)

Figure S4: Plotting the resulting number of clusters for all runs of VICatMix on simulated data when varying K_{init}. All 10 simulated datasets are generated with dimensions $N = 1000$, $P = 100$. VICatMix is run 10 times for each value of K_{init}, with $\alpha = 0.01$. The results from each run of VICatMix is plotted in green, while the mean number of clusters for each value of K_{init} is plotted in black.

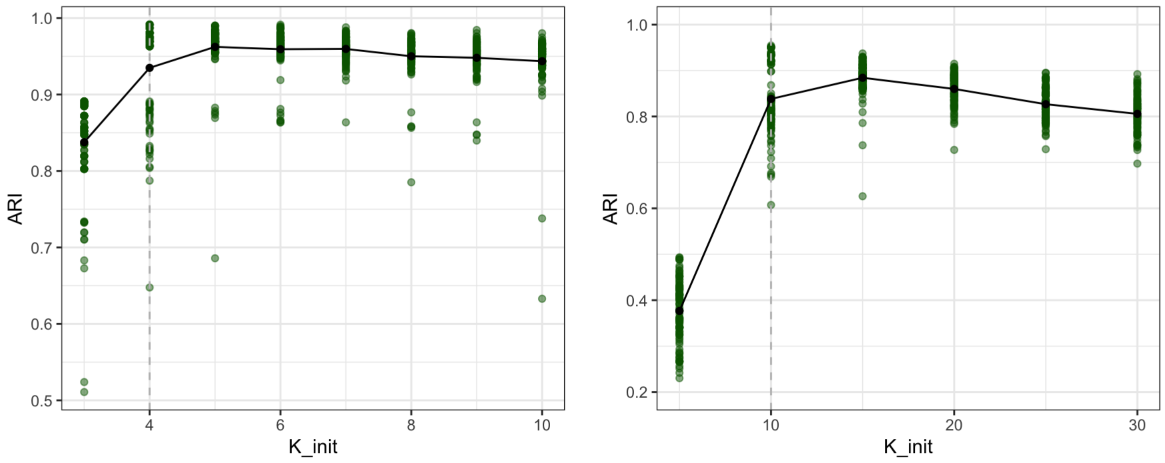

(a) 4 true clusters (sized between 100-400 points)

(b) 10 true clusters (evenly sized)

Figure S5: Plotting the resulting ARI of the clustering structure for all runs of VICatMix on simulated data when varying K_{init}. All 10 simulated datasets are generated with dimensions $N = 1000$, $P = 100$. VICatMix is run 10 times for each value of K_{init}, with $\alpha = 0.01$. The result from each run of VICatMix is plotted in green, while the mean ARI for each value of K_{init} is plotted in black.

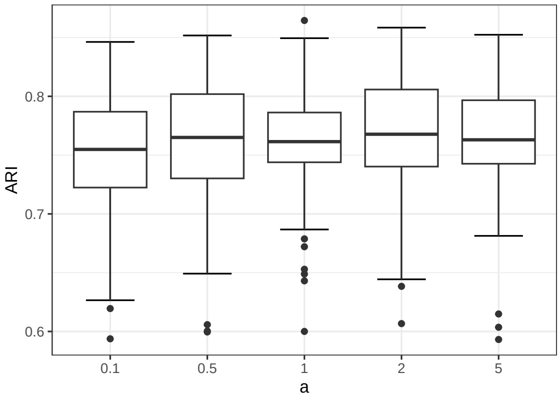

Figure S6: Simulated data with $N = 1000$, 75 relevant and 25 irrelevant variables initialised 10 times on 10 different independent datasets for each value of $a \in \{0.1, 0.5, 1, 2, 5\}$ with the resulting distribution of the ARI plotted as a boxplot.

S5.2 VICatMix-Avg simulations - additional figures

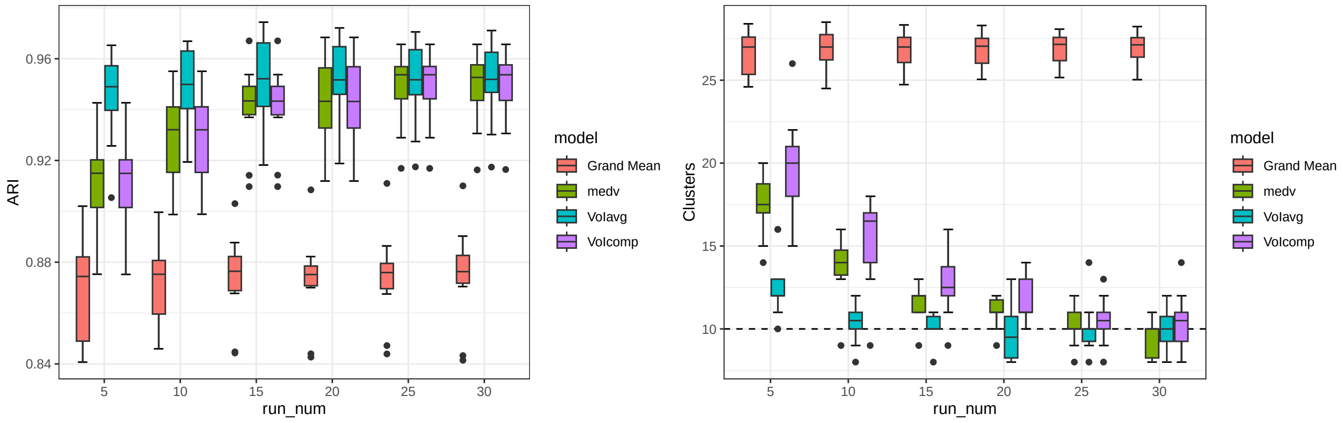

(a) (b)

Figure S7: Boxplots comparing the ARI and number of clusters of each model-averaging method across all 10 simulated datasets with the grand mean of the individual runs considered with different numbers of clustering solutions in the co-clustering matrix for Simulation 2.2.

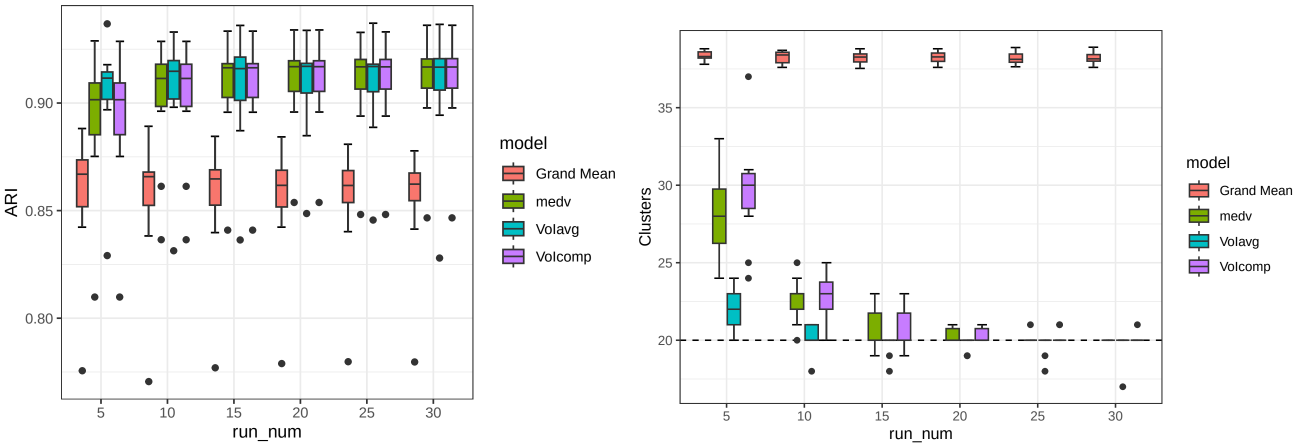

(a) (b)

Figure S8: Boxplots comparing the ARI and number of clusters of each model-averaging method across all 10 simulated datasets with the grand mean of the individual runs considered with different numbers of clustering solutions in the co-clustering matrix for Simulation 2.3.

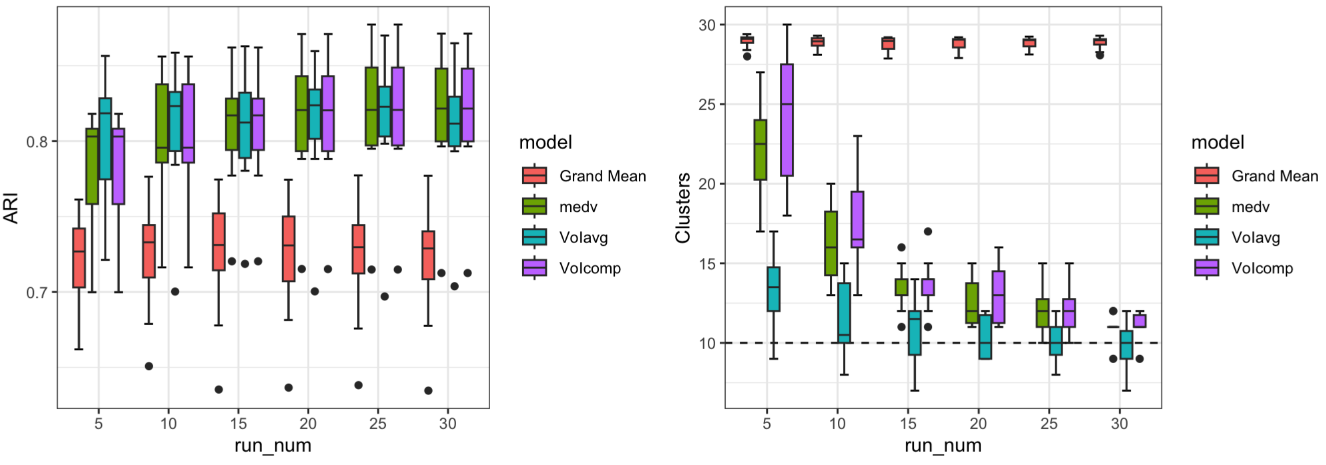

(a) (b)

Figure S9: Boxplots comparing the ARI and number of clusters of each model-averaging method across all 10 simulated datasets with the grand mean of the individual runs considered with different numbers of clustering solutions in the co-clustering matrix for Simulation 2.4.

Table S2: Table comparing the mean ARI of each summarisation method with the grand mean ARI of the runs considered in each size of co-clustering matrix (across all 10 datasets) in Simulation 2.1.

No. of Runs	Grand Mean ARI	Medv ARI	VoIavg ARI	VoIcomp ARI
5	0.815	0.892	**0.934**	0.892
10	0.842	0.924	**0.937**	0.924
15	0.826	0.934	**0.940**	0.934
20	0.836	0.940	**0.941**	0.942
25	0.841	0.939	**0.942**	0.940
30	0.845	0.940	**0.943**	0.940

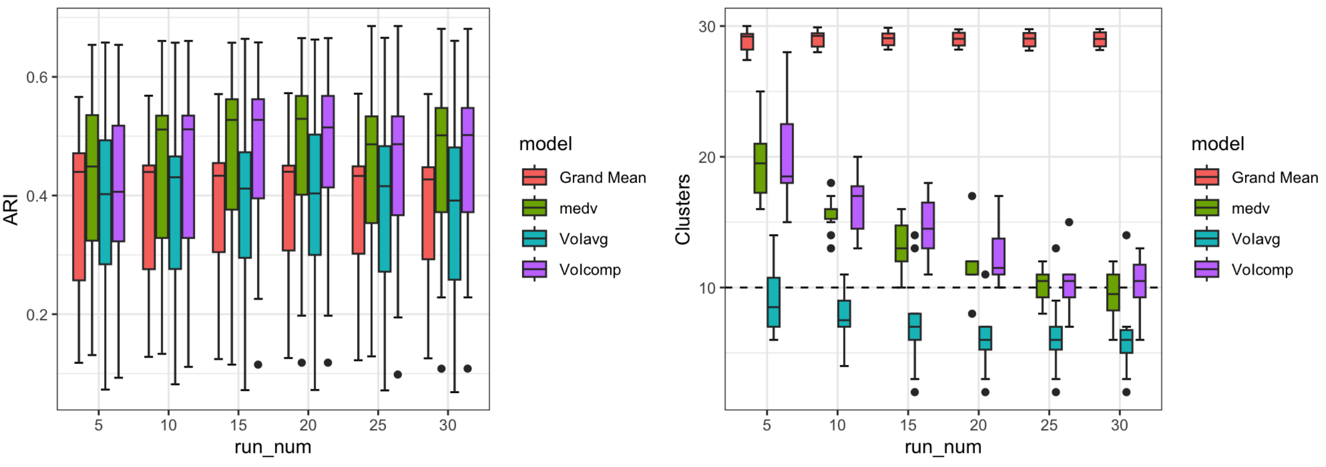

(a) (b)

Figure S10: Boxplots comparing the ARI and number of clusters of each model-averaging method across all 10 simulated datasets with the grand mean of the individual runs considered with different numbers of clustering solutions in the co-clustering matrix for Simulation 2.5.

Table S3: Table comparing the mean ARI of each summarisation method with the grand mean ARI of the runs considered in each size of co-clustering matrix (across all 10 datasets) in Simulation 2.2.

No. of Runs	Grand Mean ARI	Medv ARI	VoIavg ARI	VoIcomp ARI
5	0.870	0.910	**0.945**	0.910
10	0.871	0.929	**0.949**	0.929
15	0.874	0.941	**0.951**	0.941
20	0.873	0.943	**0.951**	0.943
25	0.874	0.949	**0.950**	0.949
30	0.875	0.949	**0.951**	0.949

Table S4: Table comparing the mean ARI of each summarisation method with the grand mean ARI of the runs considered in each size of co-clustering matrix (across all 10 datasets) in Simulation 2.3.

No. of Runs	Grand Mean ARI	Medv ARI	VoIavg ARI	VoIcomp ARI
5	0.857	0.893	**0.904**	0.893
10	0.854	0.901	**0.906**	0.901
15	0.855	**0.907**	0.907	**0.907**
20	0.854	**0.910**	0.908	**0.910**
25	0.854	**0.909**	0.908	**0.909**
30	0.854	**0.910**	0.907	**0.910**

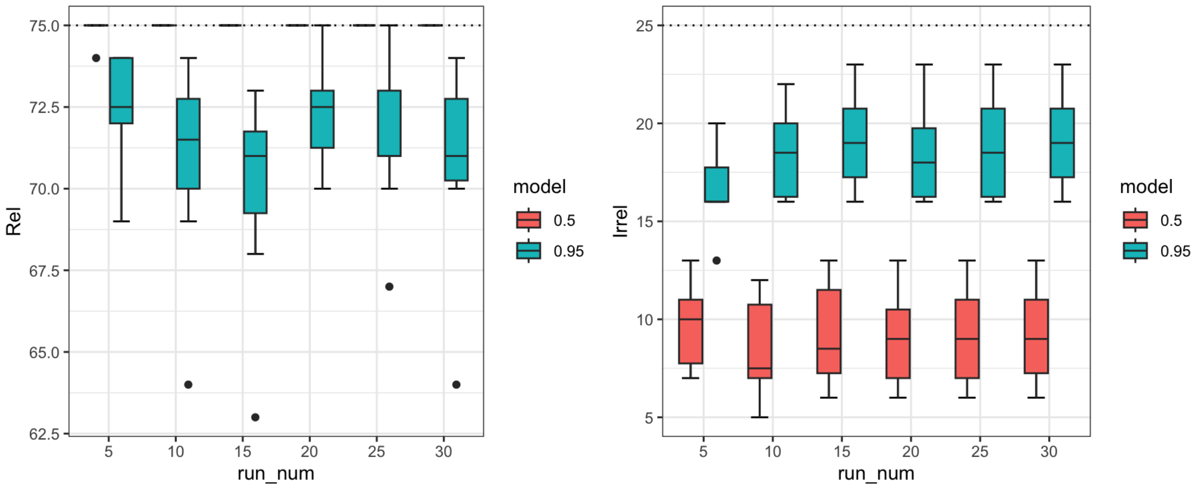

(a) (b)

Figure S11: Boxplots comparing the number of relevant and irrelevant variables discovered by thresholds $\tau = 0.5$ and $\tau = 0.95$ for finding the selected variables in Simulation 2.4 with variable selection. Note that $\tau = 0.5$ almost always finds the correct number of relevant variables, 75.

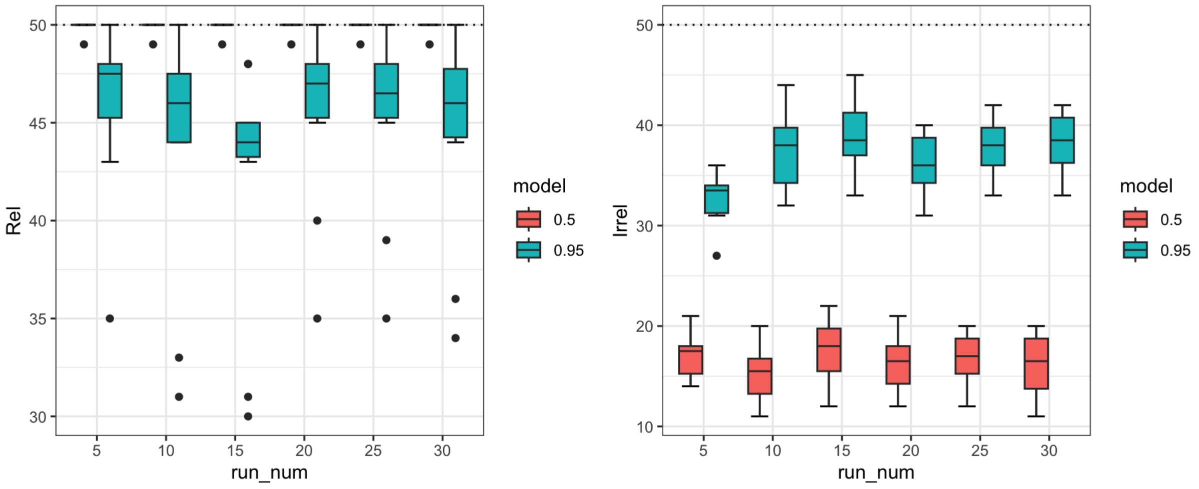

(a) (b)

Figure S12: Boxplots comparing the number of relevant and irrelevant variables discovered by thresholds $\tau = 0.5$ and $\tau = 0.95$ for finding the selected variables in Simulation 2.4 with variable selection. Note that $\tau = 0.5$ almost always finds the correct number of relevant variables, 50.

S5.2.1 Effect of varying solutions in co-clustering matrix

We additionally look at the effects of randomising which clustering solutions we use for VoI with complete linkage using 25 runs, selected from 50 different potential runs for $N = 1000$ and $P = 100$, in order to show our solution is robust to the choice of initialisations. We test this on 10 different datasets with 10 different model-averaged solutions for each dataset, and we see from Table S5 that the performance of our model-averaged clustering solution is robust to the choice of which runs are selected. All model-averaging solutions had between 10-12 clusters.

Table S5: Table comparing the mean and variance of the ARI of the model-averaged VoI runs for each simulated dataset, with the grand mean of the mean of the individual runs considered for each dataset.

Dataset	Mean Model-Averaged ARI	Variance Model-Averaged ARI	Grand Mean Individual ARI
1	0.941	0.000043	0.864
2	0.924	0.0000089	0.832
3	0.956	0.000016	0.854
4	0.954	0.0000010	0.866
5	0.934	0.0000078	0.852
6	0.934	0.000029	0.852
7	0.938	0.000022	0.864
8	0.961	0.000031	0.859
9	0.905	0.000021	0.793
10	0.932	0.000021	0.844

S5.2.2 Effect of varying initial number of clusters

Finally, as in Section S5.1.3, we look at the effects of varying the initial number of clusters, K_{init}, but now with VICatMix-Avg. As before, we simulated 10 datasets with $N = 1000$, $P = 100$ and 4 true clusters, then 10 datasets with $N = 1000$, $P = 100$ and 10 true clusters, and we ran VICatMix-Avg 5 times on each of these datasets, using VoI with complete linkage and 25 runs for the summarisation. As expected, we found that values of K_{init} greater than the true number of clusters were now able to estimate the true number of clusters much more accurately than with individual runs of VICatMix (although still with a slight increase in the number of final clusters when K_{init} was higher). We also had high and consistent levels of accuracy for values of K_{init} greater than the true number of clusters, with little difference between lower or higher values.

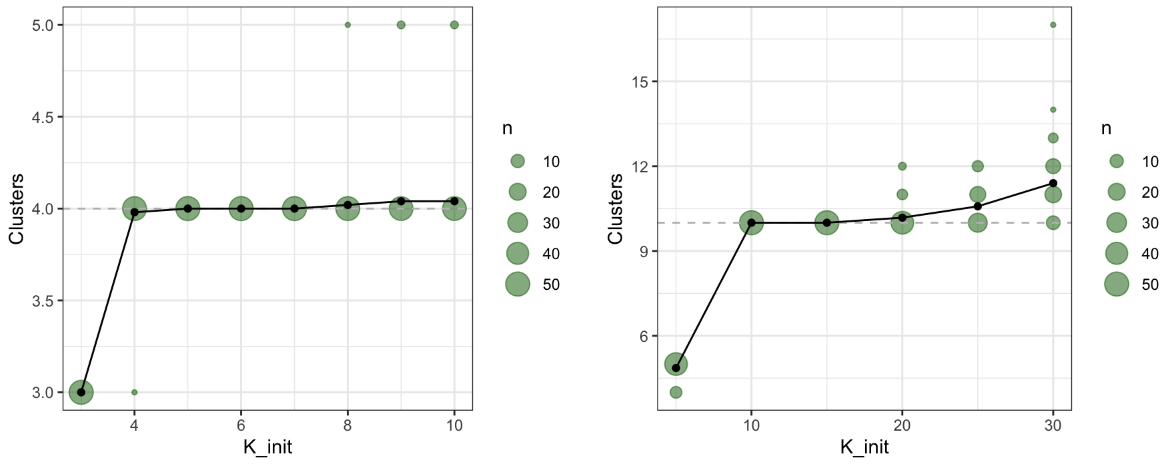

(a) 4 true clusters (sized between 100-400 points)

(b) 10 true clusters (evenly sized)

Figure S13: Plotting the resulting number of clusters for all runs of VICatMix-Avg on simulated data when varying K_{init}. All 10 simulated datasets are generated with dimensions $N = 1000$, $P = 100$. VICatMix-Avg is run 5 times for each value of K_{init}, with $\alpha = 0.01$. The results from each run of VICatMix-Avg is plotted in green, while the mean number of clusters for each value of K_{init} is plotted in black.

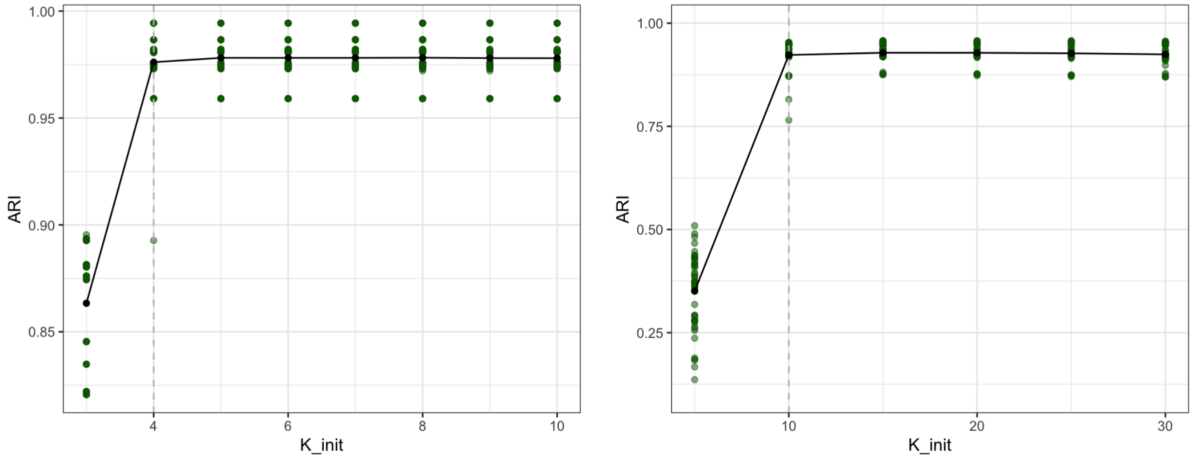

(a) 4 true clusters (sized between 100-400 points)

(b) 10 true clusters (evenly sized)

Figure S14: Plotting the resulting ARI of the clustering structure for all runs of VICatMix-Avg on simulated data when varying K_{init}. All 10 simulated datasets are generated with dimensions $N = 1000$, $P = 100$. VICatMix-Avg is run 5 times for each value of K_{init}, with $\alpha = 0.01$. The result from each run of VICatMix-Avg is plotted in green, while the mean ARI for each value of K_{init} is plotted in black.

S5.3 Comparisons to other models - set-up

Further details for the comparator methods to VICatMix, including implementation settings, are as follows:

- PReMiuM (Liverani et al., 2015): a Dirichlet process mixture model allowing for infinite components trained using Gibbs 'slice-sampler' MCMC. We use 1000 burn-in samples and 2500 sweeps. We use 'partitioning by medioids' to post-process the MCMC samples (the default value), which uses the k-medioids method (a generalisation of k-means) with the dissimilarity matrix as the distance measure; this is relatively inefficient in comparison to other post-processing methods. PReMiuM has options for two types of variable selection, based on approaches proposed by Papathomas et al.; binary variable selection, a small modification on the approach used by Chung and Dunson (Chung and Dunson, 2009) which uses a cluster specific variable selection approach by associating a binary random variable determining whether covariate j is important to mixture component c, and continuous variable selection, which performs variable selection by associating each covariate with a latent variable taking values between [0, 1] informing whether the covariate is important in supporting a mixture distribution. The continuous variable selection is similar to our method, but our latent variable is a binary random variable rather than a continuous random variable.

- Bayesian Hierarchical Clustering (BHC) (Heller and Ghahramani, 2005): implemented via R/Bioconductor (Savage et al., 2009). Performs bottom-up hierarchical clustering, where at each iteration, Bayesian hypothesis testing is used to consider which clusters should be merged. Can be interpreted as an approximate inference method for a Dirichlet process mixture model. There are no user-defined settings for the implementation of BHC in R/Bioconductor, but we note that by default, BHC performs hyperparameter tuning.

- BayesBinMix (Papastamoulis and Rattray, 2017): an MCMC implementation (with tempered MCMC chains to accelerate convergence) for Bayesian finite mixture models; the framework is almost identical to our mixture model, but a discrete prior over $1 : K_{max}$ is used to determine the number of clusters. We use 6 heated chains with heats $\{1, 0.92, 0.84, 0.76, 0.68, 0.6\}$, 500 burn-in and 2500 total MCMC sweeps, and a Poisson prior for the number of clusters per the authors' recommendations. BayesBinMix uses two algorithms - 'ECR' (Papastamoulis and Iliopoulos, 2010) and 'KL' (Stephens, 2000; Rodríguez and Walker, 2014) - to bypass the label switching issue in the MCMC output. The two algorithms give almost identical clustering solutions, although ECR is more efficient; we use ECR in our comparison.

- FlexMix (Leisch, 2004): implements an EM-algorithm under a maximum-likelihood framework for the training of finite mixture models. The *stepFlexmix* function allows for model selection by fitting a given number of models with different initialisations (we use 10) with $k = 1 : K$ clusters and finding the maximum likelihood model for each number k of clusters. The overall optimal model with the optimal number of clusters is found using penalised likelihood criteria such as the BIC or ICL criterion (Schwarz, 1978; Biernacki, Celeux, and Govaert, 2000); we found these gave the same results in all our simulations.

- Agglomerative hierarchical clustering: implemented via *hclust* in the R *stats* package with complete linkage (Kaufman and Rousseeuw, 1990; Langfelder and Horvath, 2012). We use the average silhouette function implemented in the R package *factoextra* to determine where the tree should be cut.

S5.4 Comparisons to other models - results and analysis

In these simulations, we look the ARI and the the number of clusters chosen by each model, as well as comparing run times. The results for Simulations 3.1, 3.2 and 3.3 are shown in Figures S15, S16 and S17, and Table S6.

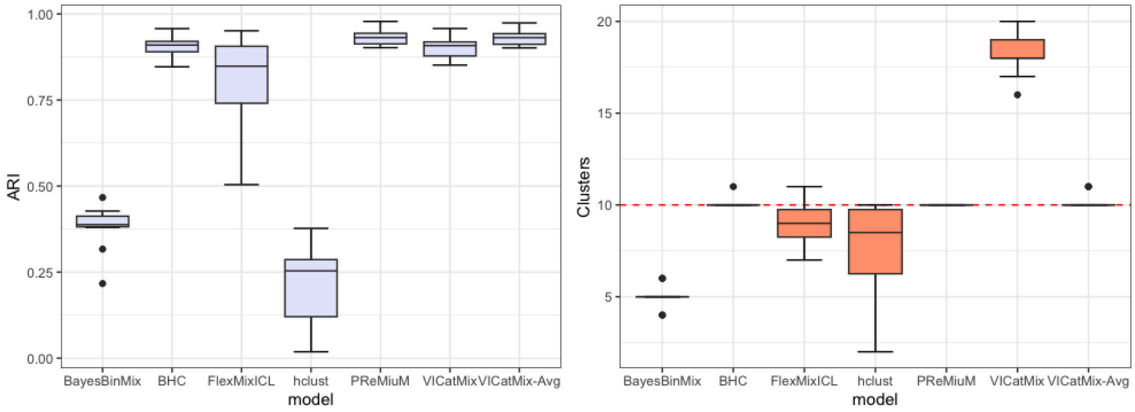

(a) (b)

Figure S15: Comparison of ARI and number of clusters found by each of the methods tested for Simulation 3.1.

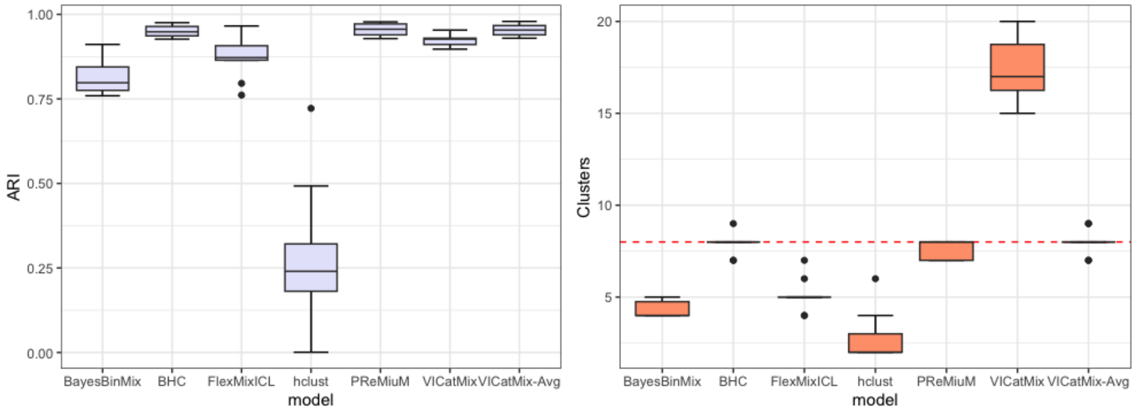

(a) (b)

Figure S16: Comparison of ARI and number of clusters found by each of the methods tested for Simulation 3.2.

In general, VICatMix-Avg was one of the best-performing models in terms of accuracy across all simulations without noisy variables, achieving ARI scores of more than 0.9 in many cases, and almost always found the correct number of clusters. VICatMix was also more time-efficient, especially as the dataset increased in size. Although individual runs of VICatMix consistently overestimated the true number of clusters as found previously, they still outperform other methods in ARI. hclust performed very poorly, BayesBinMix was lengthy and often underestimates the true number of clusters in the data, and FlexMix worked well for smaller datasets but its performance was depleted as we increased the size of the dataset and the number of true clusters in Simulation 3.3, which was also observed by the authors of BayesBinMix (Papastamoulis and Rattray, 2017). PReMiuM was the best performing alternative method to VICatMix-Avg, but being an MCMC implementation, it was slower.

Notably, BHC performed well, but much of the computational burden of BHC came from its optimisation of hyperparameters (Heller and Ghahramani, 2005). Therefore it is possible that by fixing the hyperparameters to pre-determined values, BHC could be competitive in terms of computational time to our variational algorithm; we could also optimise the equivalent hyperparameters in our model and improve accuracy at the cost of time efficiency.

Results comparing the ARI and number of clusters for Simulations 3.4 and 3.5 - the noisier datasets - are shown in Figures S18 and S19. We see that VICatMixVarSel provided a slight

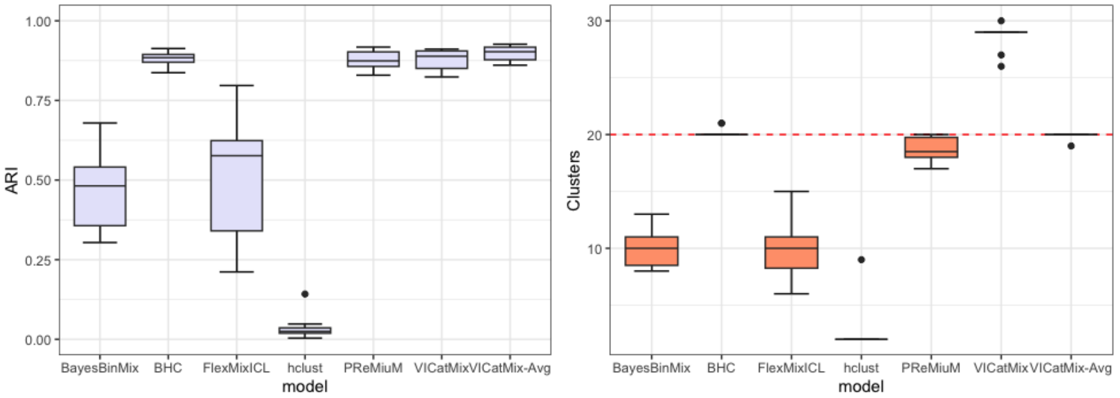

(a) (b)

Figure S17: Comparison of ARI and number of clusters found by each of the methods tested for Simulation 3.3.

Table S6: Table comparing median and quantiles of run times in seconds for each model in all simulations with no variable selection. For VICatMix-Avg, implementing the model averaging took less than a second after VICatMix was run in parallel 25 times.

Model	Simulation 3.1	Simulation 3.2	Simulation 3.3
BHC	403.8 [362.7, 443.3]	497.8 [419.1, 689.9]	2068.3 [1874.0, 2187.6]
BayesBinMix	16813.8 [16765.6, 17035.1]	16937.9 [16876.6, 17027.2]	40986.6 [40537.8, 41310.6]
FlexMix	56.9 [43.2, 96.2]	114.0 [69.8, 125.3]	379.0 [228.4, 510.6]
PReMiuM	188.1 [187.9, 190.2]	161.5 [159.2, 162.9]	623.3 [586.9, 627.9]
VICatMix	**30.9 [28.7, 33.1]**	**39.5 [32.1, 51.0]**	**103.6 [91.5, 125.9]**

increase in accuracy in terms of ARI and finding the correct number of clusters compared to VICatMix without variable selection and PReMiuM.

In Simulations 3.4 and 3.5, we additionally compared the number of relevant and irrelevant variables correctly identified by the variable selection methods using F_1 scores. For VICatMix-VarSel, we used a $\tau = 0.95$ variable selection threshold. For a variable to be considered relevant in the PReMiuM models, we looked at a threshold of 0.5 and above for the 'rho median', where 'rho' relates to the variable selection latent variable in PReMiuM taking values in [0,1] and the 'rho median' takes the median across all MCMC sweeps. We also considered a threshold of 0.95 for the rho median, analogous to our own variable selection threshold; this performed poorly compared to 0.5 so is omitted.

Table S8 shows that variable selection in VICatMix achieved the highest F_1 score in Simulation 3.4, and performed similarly to both PReMiuM variable selection methods in Simulation 3.5. Run times are seen in Table S7.

S5.5 Run-times

We saw in Section S5.4 that VICatMix was faster than other commonly used models in R. We see in Figure S20 that the run-time of our model - both with and without variable selection - approximately scaled linearly with both the number of observations (N) and the number of covariates (P). Running VICatMix (and given sufficient compute, VICatMix-Avg) is feasible for datasets with at least 20000 observations; a run takes between 10-45 minutes without variable selection and 0.5-3 hours for variable selection for $N = 20000$. Figure S21 shows that the accuracy of our model generally improved as N increases, although we also saw that the accuracy of our model in terms of ARI was depleted slightly as we increased P to $P \approx N$.

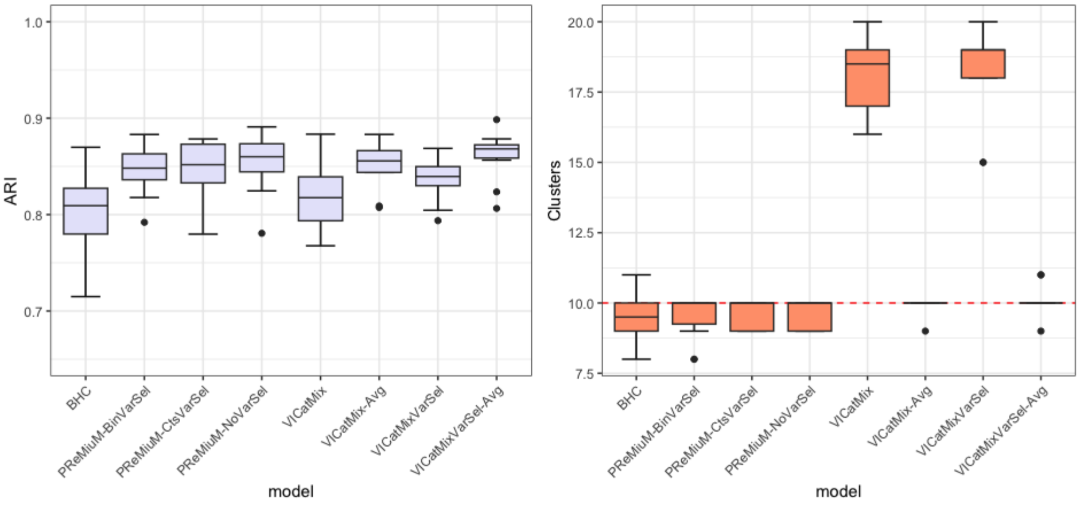

(a) (b)

Figure S18: Comparison of ARI and number of clusters found by each of the methods tested for Simulation 3.4.

Table S7: Table comparing median and quantiles of run times in seconds for each model in all simulations with variable selection. For VICatMix and VICatMixVarSel, only the times for the optimal runs are used. For VICatMix-Avg and VICatMixVarSel-Avg, implementing the model averaging took less than a second after VICatMix was run in parallel 25 times.

Model	Simulation 3.4	Simulation 3.5
BHC	377.4 [363.2, 404.6]	1780.5 [1748.5, 1791.6]
PReMiuM-BinVarSel	300.5 [290.9, 303.5]	559.8 [540.0, 592.5]
PReMIuM-CtsVarSel	363.0 [258.9, 271.8]	545.7 [511.0, 568.8]
PReMiuM-NoVarSel	184.6 [183.3, 190.2]	397.2 [384.1, 406.8]
VICatMix	**46.5 [35.6, 75.5]**	**118.5 [102.9, 153.7]**
VICatMixVarSel	**191.2 [159.3, 233.6]**	**438.6 [383.2, 613.7]**

S5.6 Categorical simulation study

We briefly illustrate that our model can be used with categorical data where variables have more than 2 possible categories in Figure S22, where we generated simulated data with 3 categories per variable. When simulating categorical data with 3 categories, we used a Dirichlet$(1, 2, 3)$ distribution as opposed to the Beta distribution used when simulating binary data.

We performed a simulation study comparing the performance of VICatMix with categorical data with BHC and PReMiuM. All of VICatMix-Avg, BHC and PReMIuM achieved extremely good results on all datasets, with ARI values between 0.98 and 1 seen in Figure S23.

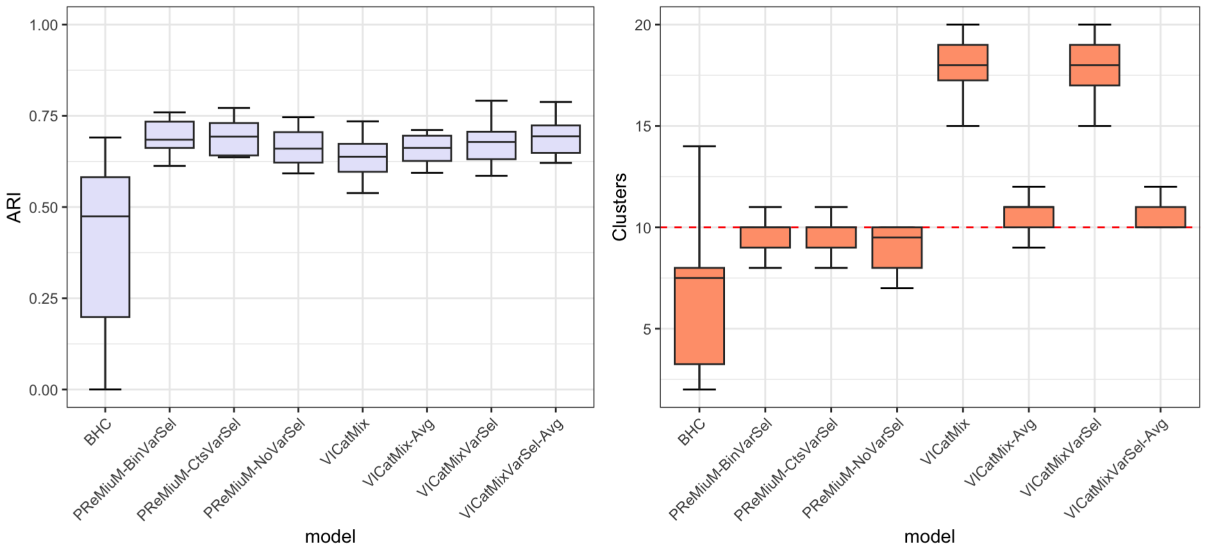

(a) (b)

Figure S19: Comparison of ARI and number of clusters found by each of the methods tested for Simulation 3.5.

Table S8: Table comparing mean F_1 scores across all datasets for variable selection methods under both Simulation 3.4 and Simulation 3.5. For VICatMixVarSel, we take the mean of the F_1 scores for only the optimal (highest ELBO) runs.

Methods	Simulation 3.4	Simulation 3.5
PReMiuM-BinVarSel	0.862	0.920
PReMiuM-CtsVarSel	0.951	**0.984**
VICatMixVarSel	0.956	0.851
VICatMixVarSel-Avg	**0.969**	0.931

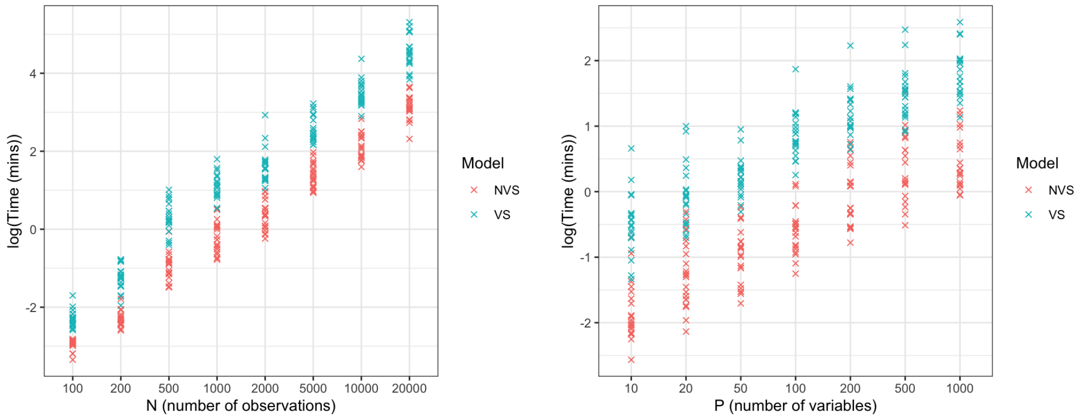

(a) Changing N where $P = 100$. (b) Changing P where $N = 1000$.

Figure S20: Graph showing how run-time of VICatMix varied as we increased N or P, the number of observations or the number of variables in the dataset in our run-times simulation study. We generated 20 independent datasets for each value of N or P with and without variable selection with 10 true clusters, and we initialised with 20 clusters. In cases with variable selection, 80% of variables are relevant. 'NVS' indicates no variable selection, 'VS' indicates variable selection.

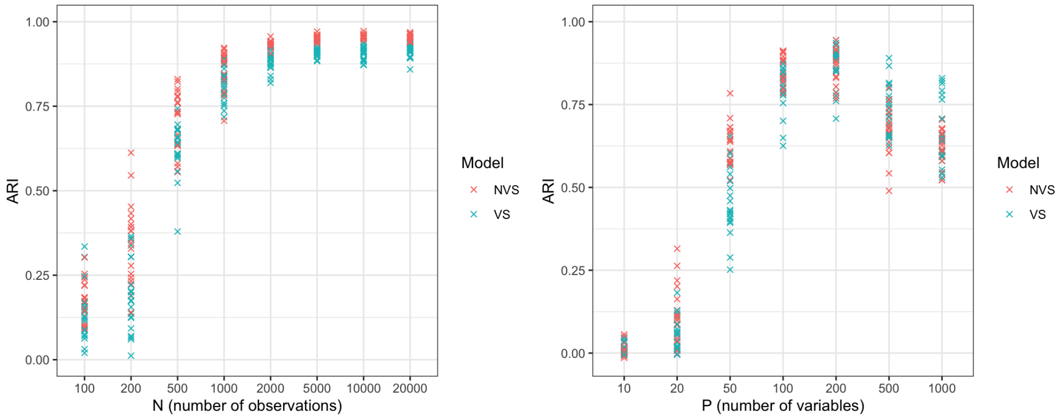

(a) Changing N where $P = 100$.

(b) Changing P where $N = 1000$.

Figure S21: Graph showing how the ARI of VICatMix varied as we increased N or P, the number of observations or the number of variables in the dataset. We generated 20 independent datasets for each value of N or P with and without variable selection with 10 true clusters, and we initialised with 20 clusters. In cases with variable selection, 80% of variables are relevant. 'NVS' indicates no variable selection, 'VS' indicates variable selection.

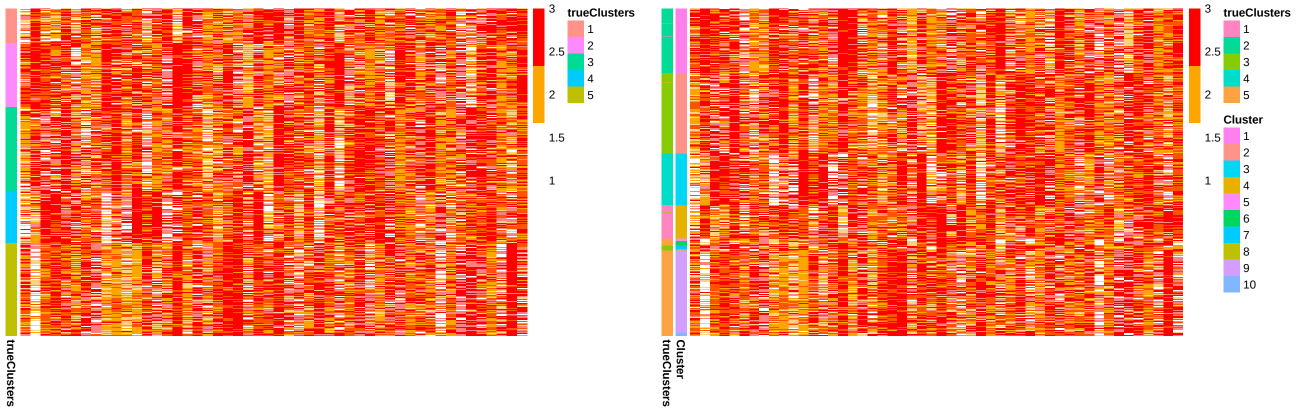

(a) Data ordered by true clusters

(b) Data ordered by VICatMix clusters

Figure S22: Heatmaps illustrating the clustering structure of categorical simulated data with $N = 1000$, $P = 50$, 5 true clusters and three categories per variable. VICatMix was run with $\alpha = 0.01$ and $K = 10$ and had an ARI of 0.910 with the true clustering structure.

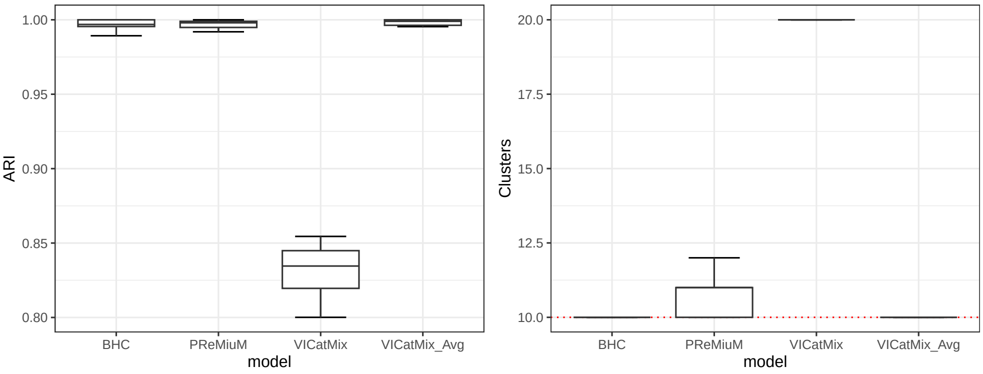

(a) Data ordered by true clusters

(b) Data ordered by VICatMix clusters

Figure S23: Boxplots comparing the distribution of ARI and number of clusters on 10 independently generated datasets after running VICatMix, VICatMix-Avg, BHC and PReMiuM on simulated data with $N = 1000$, $P = 100$, 10 evenly sized true clusters and three categories per variable. VICatMix was run with $\alpha = 0.05$ and $K = 20$ and for the VICatMix with no averaging, we took the run with the maximum ELBO.

S6 Yeast galactose data

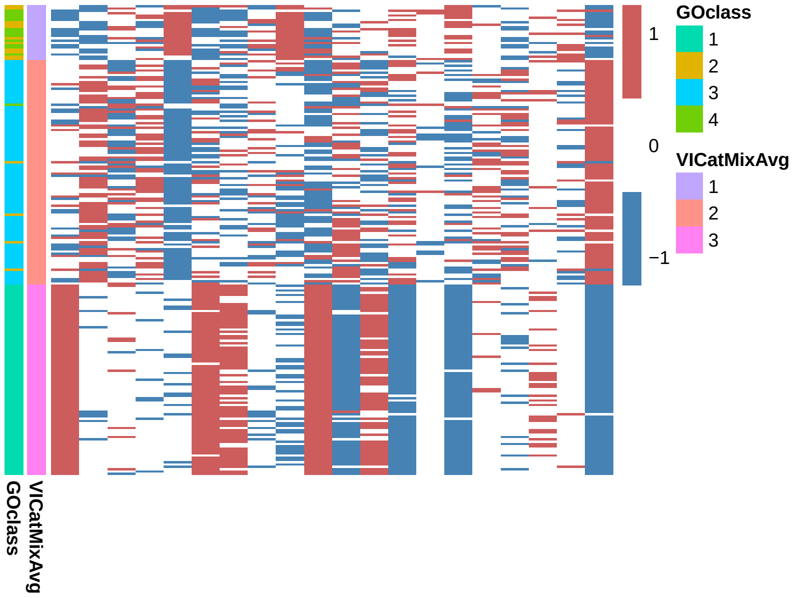

Figure S24: Heatmap of the VICatMix-Avg clustering structure on the yeast galactose data compared with the GO labelling when K=4.

S7 Acute myeloid leukaemia (AML)

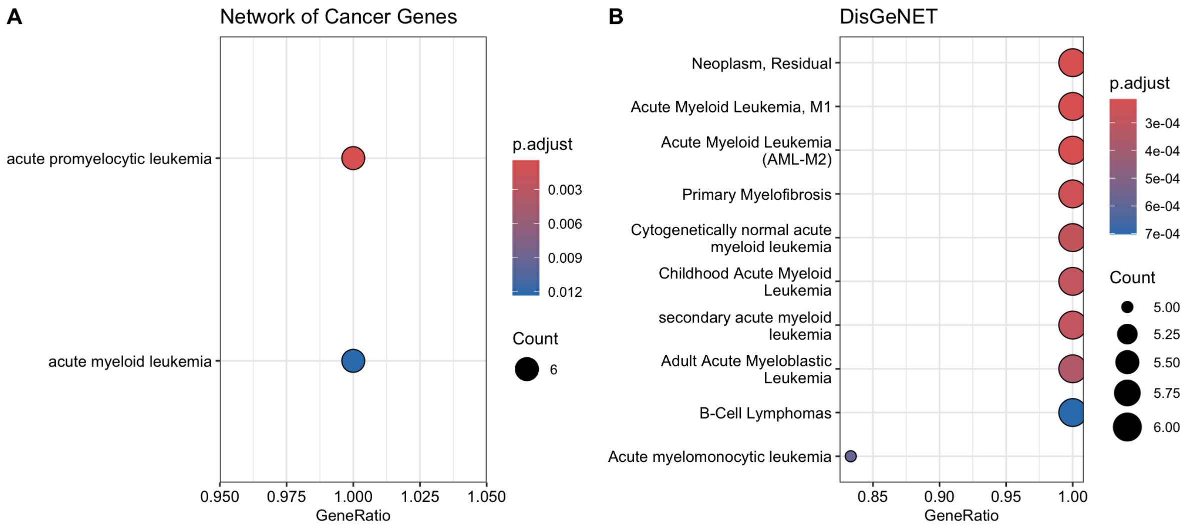

Figure S25: Dotplots visualising over-representation analysis for 6 selected genes for the AML dataset using gene-disease annotations from the Network of Cancer Genes and DisGeNET.

S8 Pan-cancer cluster-of-clusters analysis with K = 15 - additional figures

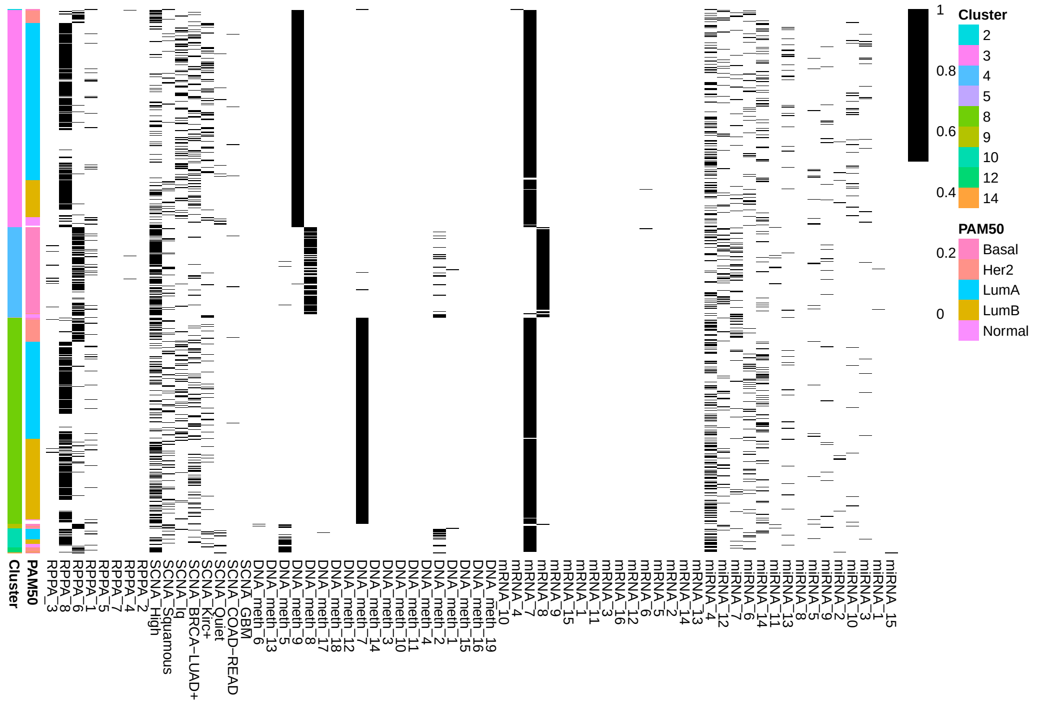

Figure S26: A heatmap showing the VICatMix-Avg clustering of the Matrix of Clusters for the BRCA (breast invasive carcinoma) samples in our pan-cancer data, comparing the clusters to known PAM50 subtypes.

**Tissue**	**1**	**2**	**3**	**4**	**5**	**6**	**7**	**8**	**9**	**10**	**11**	**12**	**13**	**14**	**15**
**BLCA**	0	21	0	0	0	0	0	0	97	1	0	0	0	1	0
**BRCA**	0	1	333	138	1	0	0	316	7	29	0	8	0	1	0
**COAD**	0	0	0	0	0	0	182	0	0	0	0	0	0	0	0
**GBM**	0	0	0	0	0	0	0	0	3	0	0	0	189	3	0
**HNSC**	0	302	0	0	0	0	0	0	2	0	0	0	0	1	0
**KIRC**	0	0	0	0	471	0	0	0	3	0	0	0	1	0	0
**LAML**	0	0	0	0	0	0	0	0	0	0	0	0	0	0	161
**LUAD**	255	6	0	0	0	1	0	0	8	0	0	0	0	0	0
**LUSC**	16	206	0	0	0	0	0	0	16	0	0	0	0	0	0
**OV**	0	0	0	0	0	0	0	0	1	0	327	0	0	1	0
**READ**	0	0	0	0	0	0	73	0	0	0	0	0	0	0	0
**UCEC**	0	0	0	0	0	342	1	0	2	0	0	0	0	0	0

Table S9: Cluster assignments by tissue for the pan-cancer data where K=15. A list of the abbreviations used for TCGA tissue types can be found at the link `https://gdc.cancer.gov/resources-tcga-users/tcga-code-tables/tcga-study-abbreviations` (accessed 23 Oct 2024).

S9 Pan-cancer cluster-of-clusters analysis with K = 40

We illustrate here the performance of our model when considering $K = 40$ in each run of VICatMix in order to investigate sub-clustering structures within tissues of origin. We see in Figures S27 and S28 that once again the clusters clearly corresponded with tissue of origin, and many tissues were subdivided into multiple clusters. For example, Cluster 33 corresponded exactly with LAML (acute myeloid leukaemia) samples; Clusters 7, 17 and 19 corresponded with UCEC (uterine corpus endometrial carcinoma) samples; and Cluster 8 corresponded precisely with a mixed COAD (colon adenocarcinoma)/READ (rectum adenocarcinoma) cluster. Both are colorectal adenocarcinomas and have been previously reported to have very similar expression features at the molecular level - for example, a study by TCGA suggested that non-hypermutated adenocarcinomas of the colon and rectum were not distinguishable at the genomic level (The Cancer Genome Atlas Network, 2012), and analysis in the Molecular Epidemiology of Colorectal Cancer (MECC) study (Sanz-Pamplona et al., 2011) suggested that there were minimal statistically significant differentially expressed genes between different tumour locations in colorectal cancer patients.

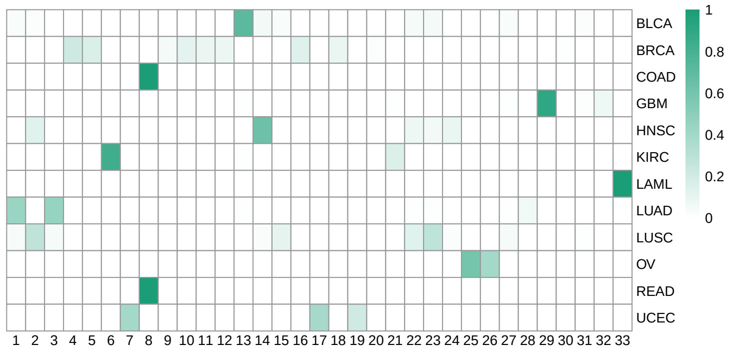

Figure S27: A heatmap showing the correspondence between clusters produced by our model and tissues of origin. A darker cell colour in row i indicates a higher percentage of samples from tissue i are in the given cluster j.

Notably, when we considered the model in Section 5.4 of the main paper with $K = 15$ we saw that clusters within tissue types were combined. This suggests that VICatMix is able to detect a hierarchical structure within data; with the number of clusters restricted, clusters were generally separated by tissue type.

Looking at the BRCA (breast invasive carcinoma) samples in depth (Figure S29), we found that the Basal-like samples were again clearly separated, with 133/141 Basal samples falling into this cluster. Furthermore, Cluster 9 contained over half of the HER2-enriched samples (34/66). This suggests that our clustering method is able to identify and separate another clinically relevant subtype. Other clusters were mostly mixtures of Luminal A and Luminal B samples; it could be of biological interest to look into why these samples were placed in different DNA methylation and somatic copy number clusters.

VICatMix-Avg's ability to identify these PAM50 BRCA subtypes motivates its application to the identification of other cancer subtypes. For example, ovarian cancer samples were divided into two clusters, seemingly based on somatic copy number clustering where samples are in the SCNA-High and SCNA-Squamous clusters. Serous ovarian carcinomas are known to have extensive copy number alterations (where few are recurrent) and highly complex genomic profiles (Macintyre et al., 2018; Hoadley et al., 2014), making it difficult to investigate the mutational processes leading to copy number changes. Little progression has been made in identifying robust clinical subtypes, and gene expression subtypes proposed thus far, such as a 4-subtype classification by the TCGA, have been found to lack robustness across other independent cohorts of patients (Verhaak et al., 2012; Chen et al., 2018; The Cancer Genome Atlas Research Network, 2011). We found little correlation between the two clusters and the 4 gene expression subtypes. It could therefore be interesting to investigate what is driving these ovarian cancer samples to fall into different SCNA (somatic copy

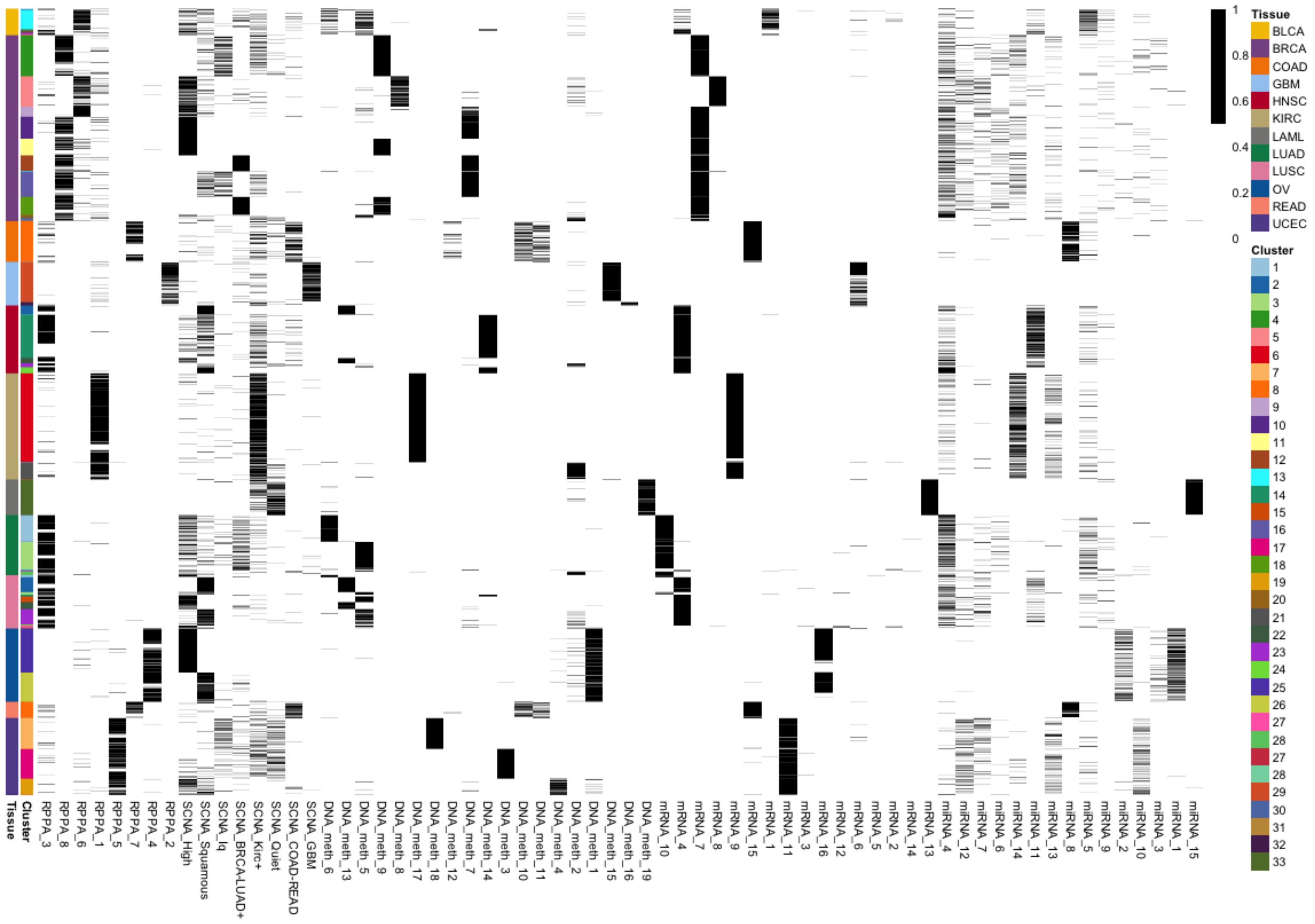

Figure S28: A heatmap showing the VICatMix-Avg clustering of the Matrix of Clusters for our pan-cancer data, comparing the clusters to the tissue of origin.

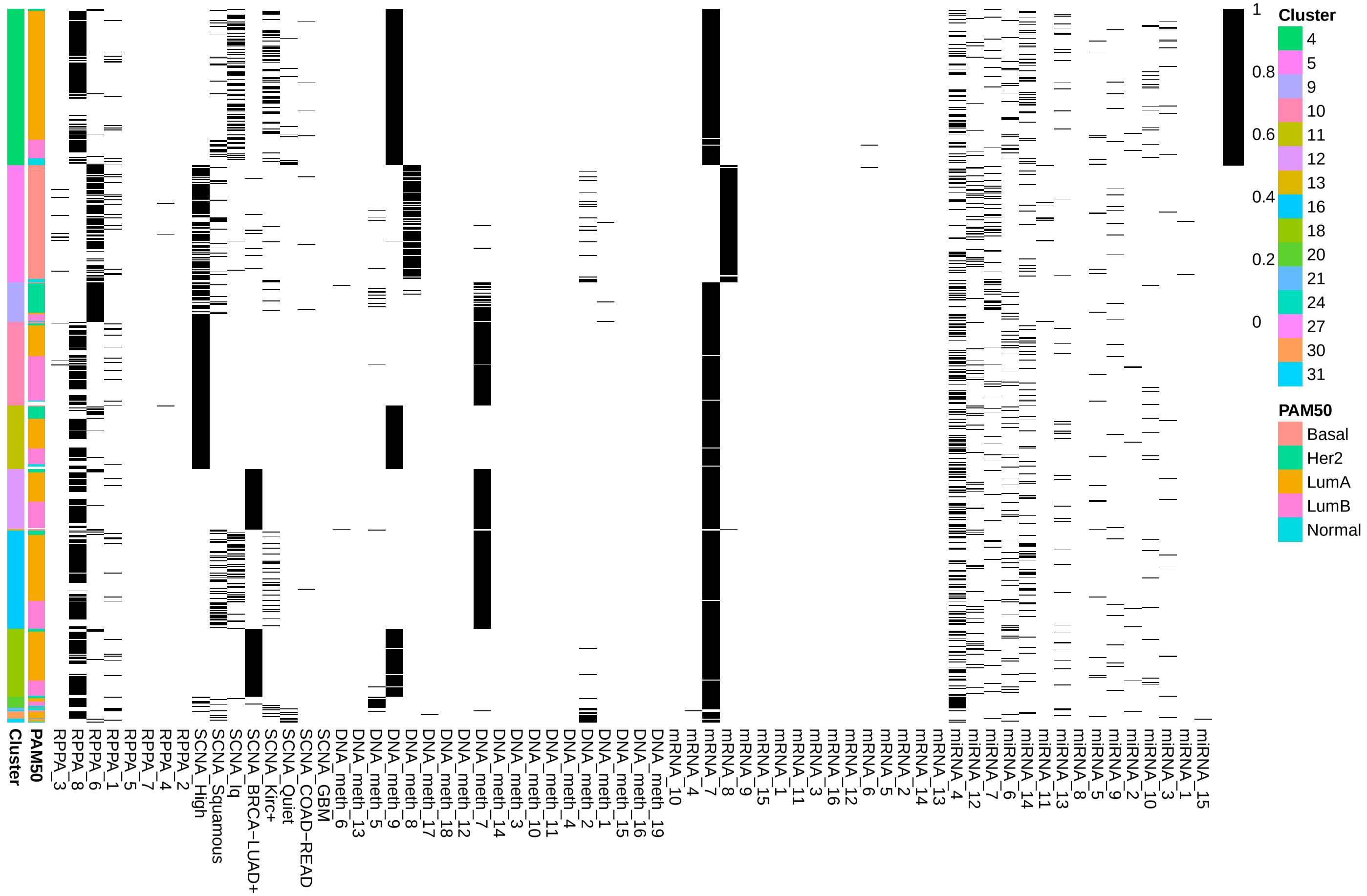

Figure S29: A heatmap showing the clustering of all breast cancers in our pan-cancer data, comparing our optimal VICatMix clusters to the PAM50 subtype from Berger et al. (2018).

number) clusters under the hierarchical clustering analysis by Hoadley et al. which may motivate the identification of clinically relevant ovarian cancer genomic subtypes.

References

Berger, Ashton C., Anil Korkut, Rupa S. Kanchi, et al. (Apr. 2018). "A Comprehensive Pan-Cancer Molecular Study of Gynecologic and Breast Cancers". In: *Cancer Cell* 33.4, 690–705.e9. ISSN: 1535-6108. DOI: 10.1016/j.ccell.2018.03.014. URL: http://dx.doi.org/10.1016/j.ccell.2018.03.014.

Biernacki, C., Gilles Celeux, and Gérard Govaert (July 2000). "Assessing a mixture model for clustering with the integrated completed likelihood". In: *IEEE Transactions on Pattern Analysis and Machine Intelligence* 22.7, pp. 719–725. ISSN: 0162-8828. DOI: 10.1109/34.865189.

Binder, D. A. (1978). "Bayesian cluster analysis". In: *Biometrika* 65.1, pp. 31–38. ISSN: 1464-3510. DOI: 10.1093/biomet/65.1.31.

Chaturvedi, Anil, Paul E. Green, and J. Douglas Caroll (Jan. 2001). "K-modes Clustering". In: *Journal of Classification* 18.1, pp. 35–55. ISSN: 1432-1343. DOI: 10.1007/s00357-001-0004-3.

Chaumeny, Yannis, Johan van der Molen Moris, Anthony C. Davison, et al. (2022). "Bayesian nonparametric mixture inconsistency for the number of components: How worried should we be in practice?" In: DOI: 10.48550/ARXIV.2207.14717. URL: https://arxiv.org/abs/2207.14717.

Chen, Gregory M., Lavanya Kannan, Ludwig Geistlinger, et al. (Oct. 2018). "Consensus on Molecular Subtypes of High-Grade Serous Ovarian Carcinoma". In: *Clinical Cancer Research* 24.20, pp. 5037–5047. ISSN: 1557-3265. DOI: 10.1158/1078-0432.ccr-18-0784.

Chung, Yeonseung and David B. Dunson (Dec. 2009). "Nonparametric Bayes Conditional Distribution Modeling With Variable Selection". In: *Journal of the American Statistical Association* 104.488, pp. 1646–1660. ISSN: 1537-274X. DOI: 10.1198/jasa.2009.tm08302.

Crook, Oliver M., Laurent Gatto, and Paul D. W. Kirk (Dec. 2019). "Fast approximate inference for variable selection in Dirichlet process mixtures, with an application to pan-cancer proteomics". In: *Statistical Applications in Genetics and Molecular Biology* 18.6. DOI: 10.1515/sagmb-2018-0065.

Fritsch, Arno and Katja Ickstadt (June 2009). "Improved criteria for clustering based on the posterior similarity matrix". In: *Bayesian Analysis* 4.2. ISSN: 1936-0975. DOI: 10.1214/09-ba414.

Heller, Katherine A. and Zoubin Ghahramani (2005). "Bayesian hierarchical clustering". In: *Proceedings of the 22nd international conference on Machine learning - ICML '05*. ICML '05. ACM Press. DOI: 10.1145/1102351.1102389.

Hoadley, Katherine A., Christina Yau, Denise M. Wolf, et al. (Aug. 2014). "Multiplatform Analysis of 12 Cancer Types Reveals Molecular Classification within and across Tissues of Origin". In: *Cell* 158.4, pp. 929–944. ISSN: 0092-8674. DOI: 10.1016/j.cell.2014.06.049.

Kaufman, Leonard and Peter J. Rousseeuw (Mar. 1990). *Finding Groups in Data: An Introduction to Cluster Analysis*. Wiley. ISBN: 9780470316801. DOI: 10.1002/9780470316801. URL: http://dx.doi.org/10.1002/9780470316801.

Langfelder, Peter and Steve Horvath (Mar. 2012). "Fast R functions for robust correlations and hierarchical clustering". en. In: *J. Stat. Softw.* 46.11.

Leisch, Friedrich (2004). "FlexMix: A General Framework for Finite Mixture Models and Latent Class Regression in R". In: *Journal of Statistical Software* 11.8. ISSN: 1548-7660. DOI: 10.18637/jss.v011.i08.

Lijoi, Antonio, Igor Prünster, and Giovanni Rebaudo (Feb. 2022). "Flexible clustering via hidden hierarchical Dirichlet priors". In: *Scandinavian Journal of Statistics* 50.1, pp. 213–234. ISSN: 1467-9469. DOI: 10.1111/sjos.12578.

Liverani, Silvia, David I. Hastie, Lamiae Azizi, et al. (2015). "PReMiuM: An R Package for Profile Regression Mixture Models Using Dirichlet Processes". In: *Journal of Statistical Software* 64.7. ISSN: 1548-7660. DOI: 10.18637/jss.v064.i07.

Macintyre, Geoff, Teodora E. Goranova, Dilrini De Silva, et al. (Aug. 2018). "Copy number signatures and mutational processes in ovarian carcinoma". In: *Nature Genetics* 50.9, pp. 1262–1270. ISSN: 1546-1718. DOI: 10.1038/s41588-018-0179-8.

Medvedovic, Mario, Ka Yee Yeung, and Roger E. Bumgarner (Feb. 2004). "Bayesian mixture model based clustering of replicated microarray data". In: *Bioinformatics* 20.8, pp. 1222–1232. ISSN: 1367-4803. DOI: 10.1093/bioinformatics/bth068.

Meilă, Marina (May 2007). "Comparing clusterings—an information based distance". In: *Journal of Multivariate Analysis* 98.5, pp. 873–895. ISSN: 0047-259X. DOI: 10.1016/j.jmva.2006.11.013.

Papastamoulis, Panagiotis and George Iliopoulos (Jan. 2010). "An Artificial Allocations Based Solution to the Label Switching Problem in Bayesian Analysis of Mixtures of Distributions". In: *Journal of Computational and Graphical Statistics* 19.2, pp. 313–331. ISSN: 1537-2715. DOI: 10.1198/jcgs.2010.09008. URL: http://dx.doi.org/10.1198/jcgs.2010.09008.

Papastamoulis, Panagiotis and Magnus Rattray (2017). "BayesBinMix: an R Package for Model Based Clustering of Multivariate Binary Data". In: *The R Journal* 9.1, pp. 403–420. DOI: 10.32614/RJ-2017-022.

Papathomas, Michail, John Molitor, Clive Hoggart, et al. (July 2012). "Exploring Data From Genetic Association Studies Using Bayesian Variable Selection and the Dirichlet Process: Application to Searching for Gene × Gene Patterns". In: *Genetic Epidemiology* 36.6, pp. 663–674. ISSN: 1098-2272. DOI: 10.1002/gepi.21661.

Rasmussen, Carl, Bernard J. de la Cruz, Zoubin Ghahramani, et al. (Oct. 2009). "Modeling and Visualizing Uncertainty in Gene Expression Clusters Using Dirichlet Process Mixtures". In: *IEEE/ACM Transactions on Computational Biology and Bioinformatics* 6.4, pp. 615–628. ISSN: 2374-0043. DOI: 10.1109/tcbb.2007.70269.

Rastelli, Riccardo and Nial Friel (Oct. 2017). "Optimal Bayesian estimators for latent variable cluster models". In: *Statistics and Computing* 28.6, pp. 1169–1186. ISSN: 1573-1375. DOI: 10.1007/s11222-017-9786-y.

Rodríguez, Carlos E. and Stephen G. Walker (Jan. 2014). "Label Switching in Bayesian Mixture Models: Deterministic Relabeling Strategies". In: *Journal of Computational and Graphical Statistics* 23.1, pp. 25–45. ISSN: 1537-2715. DOI: 10.1080/10618600.2012.735624. URL: http://dx.doi.org/10.1080/10618600.2012.735624.

Sanz-Pamplona, Rebeca, David Cordero, Antonio Berenguer, et al. (Nov. 2011). "Gene Expression Differences between Colon and Rectum Tumors". In: *Clinical Cancer Research* 17.23, pp. 7303–7312. ISSN: 1557-3265. DOI: 10.1158/1078-0432.ccr-11-1570.

Savage, Richard S, Katherine A. Heller, Yang Xu, et al. (Aug. 2009). "R/BHC: fast Bayesian hierarchical clustering for microarray data". In: *BMC Bioinformatics* 10.1. ISSN: 1471-2105. DOI: 10.1186/1471-2105-10-242.

Schwarz, Gideon (Mar. 1978). "Estimating the Dimension of a Model". In: *The Annals of Statistics* 6.2. ISSN: 0090-5364. DOI: 10.1214/aos/1176344136.

Stephens, Matthew (Nov. 2000). "Dealing With Label Switching in Mixture Models". In: *Journal of the Royal Statistical Society Series B: Statistical Methodology* 62.4, pp. 795–809. ISSN: 1467-9868. DOI: 10.1111/1467-9868.00265. URL: http://dx.doi.org/10.1111/1467-9868.00265.

The Cancer Genome Atlas Network (July 2012). "Comprehensive molecular characterization of human colon and rectal cancer". In: *Nature* 487.7407, pp. 330–337. ISSN: 1476-4687. DOI: 10.1038/nature11252.

The Cancer Genome Atlas Research Network (June 2011). "Integrated genomic analyses of ovarian carcinoma". In: *Nature* 474.7353, pp. 609–615. ISSN: 1476-4687. DOI: 10.1038/nature10166.

Verhaak, Roel G.W., Pablo Tamayo, Ji-Yeon Yang, et al. (Dec. 2012). "Prognostically relevant gene signatures of high-grade serous ovarian carcinoma". In: *Journal of Clinical Investigation.* ISSN: 0021-9738. DOI: 10.1172/jci65833.

Wade, Sara and Zoubin Ghahramani (June 2018). "Bayesian Cluster Analysis: Point Estimation and Credible Balls (with Discussion)". In: *Bayesian Analysis* 13.2. ISSN: 1936-0975. DOI: 10.1214/17-ba1073.
